# Unravelling the limb regeneration mechanisms of *Polypedates maculatus*, a sub-tropical frog, by transcriptomics

**DOI:** 10.1186/s12864-023-09205-8

**Published:** 2023-03-16

**Authors:** Cuckoo Mahapatra, Pranati Naik, Sumanta Kumar Swain, Pratyush Paradarsita Mohapatra

**Affiliations:** 1 P.G. Department of Zoology, Maharaja Sriram Chandra Bhanja Deo University, Baripada, Odisha 757003 India; 2 Zoological Survey of India, Indian Museum Complex, JL Road, Kolkata, West Bengal 700016 India

**Keywords:** Myeloid cells, Proteoglycans, Collagens, Methyltransferases, In situ hybridisation

## Abstract

**Background:**

Regeneration studies help to understand the strategies that replace a lost or damaged organ and provide insights into approaches followed in regenerative medicine and engineering. Amphibians regenerate their limbs effortlessly and are indispensable models to study limb regeneration. *Xenopus* and axolotl are the key models for studying limb regeneration but recent studies on non-model amphibians have revealed species specific differences in regeneration mechanisms.

**Results:**

The present study describes the de novo transcriptome of intact limbs and three-day post-amputation blastemas of tadpoles and froglets of the Asian tree frog *Polypedates maculatus*, a non-model amphibian species commonly found in India. Differential gene expression analysis between early tadpole and froglet limb blastemas discovered species-specific novel regulators of limb regeneration. The present study reports upregulation of proteoglycans, such as epiphycan, chondroadherin, hyaluronan and proteoglycan link protein 1, collagens 2,5,6, 9 and 11, several tumour suppressors and methyltransferases in the *P. maculatus* tadpole blastemas. Differential gene expression analysis between tadpole and froglet limbs revealed that in addition to the expression of larval-specific haemoglobin and glycoproteins, an upregulation of cysteine and serine protease inhibitors and downregulation of serine proteases, antioxidants, collagenases and inflammatory genes in the tadpole limbs were essential for creating an environment that would support regeneration. Dermal myeloid cells were GAG+, EPYC+, INMT+, LEF1+ and SALL4+ and seemed to migrate from the unamputated regions of the tadpole limb to the blastema. On the other hand, the myeloid cells of the froglet limb blastemas were few and probably contributed to sustained inflammation resulting in healing.

**Conclusions:**

Studies on non-model amphibians give insights into alternate tactics for limb regeneration which can help devise a plethora of methods in regenerative medicine and engineering.

**Supplementary Information:**

The online version contains supplementary material available at 10.1186/s12864-023-09205-8.

## Background

Regeneration studies help to identify strategies that create a pro-regenerative environment and restore damaged and diseased organs. They provide valuable inputs for enhancing regenerative outcomes in regeneration-deficient organisms like humans [1] and design strategies for regenerative medicine [2]. Amphibians are well-exploited for studying limb regeneration and recent transcriptomic studies on axolotl [3, 4], newts [5, 6], and *Xenopus* [7] have elucidated the signalling pathways, molecules and transcription factors involved during limb regeneration. Single-cell RNA-seq has also transformed the study by identifying novel cells involved during regeneration [4, 7, 8] but lag in giving definite answers to an overwhelming question - why do some limbs regenerate while others do not? Animals show variability in regenerating tissue or organ- some tissues show high regenerative potential while others fail to regenerate, even in the same individual. Normally, younger tissues regenerate seamlessly as compared to older tissues. The underlying reasons for the difference in regenerative capacity can range from the availability of stem or progenitor cells, dedifferentiation and transdifferentiation potential, presence of regeneration-specific genes, DNA methylation and immune response [9, 10]. Limb regeneration mechanisms has been elucidated in model organisms but it has not been possible to regenerate a complex organ such as a limb in regeneration-deficient animals. Part of the problem also lies in the fact that regenerative strategies have not been explored across the animal kingdom consequently restricting our understanding of the process [11]. Even in the case of amphibians, species-specific differences in the mechanisms [12] makes limb regeneration a complex process to decipher.

One way to address this problem would be to compare the conditions that inhibit regeneration with those that lead to successful regeneration. Regenerative strategies in the regeneration-capable amphibian species [6, 13, 14], regeneration capable and normal or experimentally induced regeneration deficient stages of *Xenopus* [15–17] or axolotl [18, 19] have been compared. Anuran amphibians become an inimitable choice to do comparative studies as they successfully regenerate limbs as tadpoles while the metamorphosed juveniles and adults do not regrow their amputated limbs [20]. Aztekin et al. [21] compared the single-cell transcriptome of regenerating and regeneration incompetent stages of tadpoles of *Xenopus* and described Noggin as important for regeneration incompetency. The decline in regenerative ability is due to the intrinsic properties of limb cells rather than the extrinsic factors [10, 22, 23]. However, it has always assumed that regeneration-incompetent organs possess a latent potential that can be activated by specific morphogenetic agents and cause regeneration [23, 24]. But, experiments to enhance the regeneration capacity of froglets were not able to regenerate a fully functional limb [14, 25] and crucial components for regeneration are yet to discovered.

In the present study, we attempted to elucidate the transcriptome of homeostatic hind limbs and 3 days post-amputation hind limb blastemas of tadpole and froglet of an unsequenced frog, *Polypedates maculatus* by de novo RNA sequencing. The Asian tree frog, *P. maculatus,* is commonly available in the Indian sub-continent that breeds during monsoons and lays eggs in foam nests [26]. Following transcriptome analysis, we experimentally validated our transcriptome’s accuracy by analysing the expression of transcripts by real-time PCR. Differential gene expression (DGE) analysis between intact tadpole and froglet limbs helped to understand the molecular repertoire of limbs that supports regeneration vis-a-vis one that does not. DGE analysis between intact limbs and blastemas of tadpoles or froglets identified upregulated and downregulated genes during regeneration or healing, respectively. DGE analysis between hind limb blastemas of froglets and tadpoles compared the molecular repertoire between successful and unsuccessful regeneration. Finally, the expression of selected differentially expressed genes was analysed by Real time-PCR and in situ hybridisation at different time points in the limb blastemas of tadpoles and froglets.

## Results

### Limb regeneration in tadpoles and healing in froglets of *P. maculatus*

**Fig. 1:**
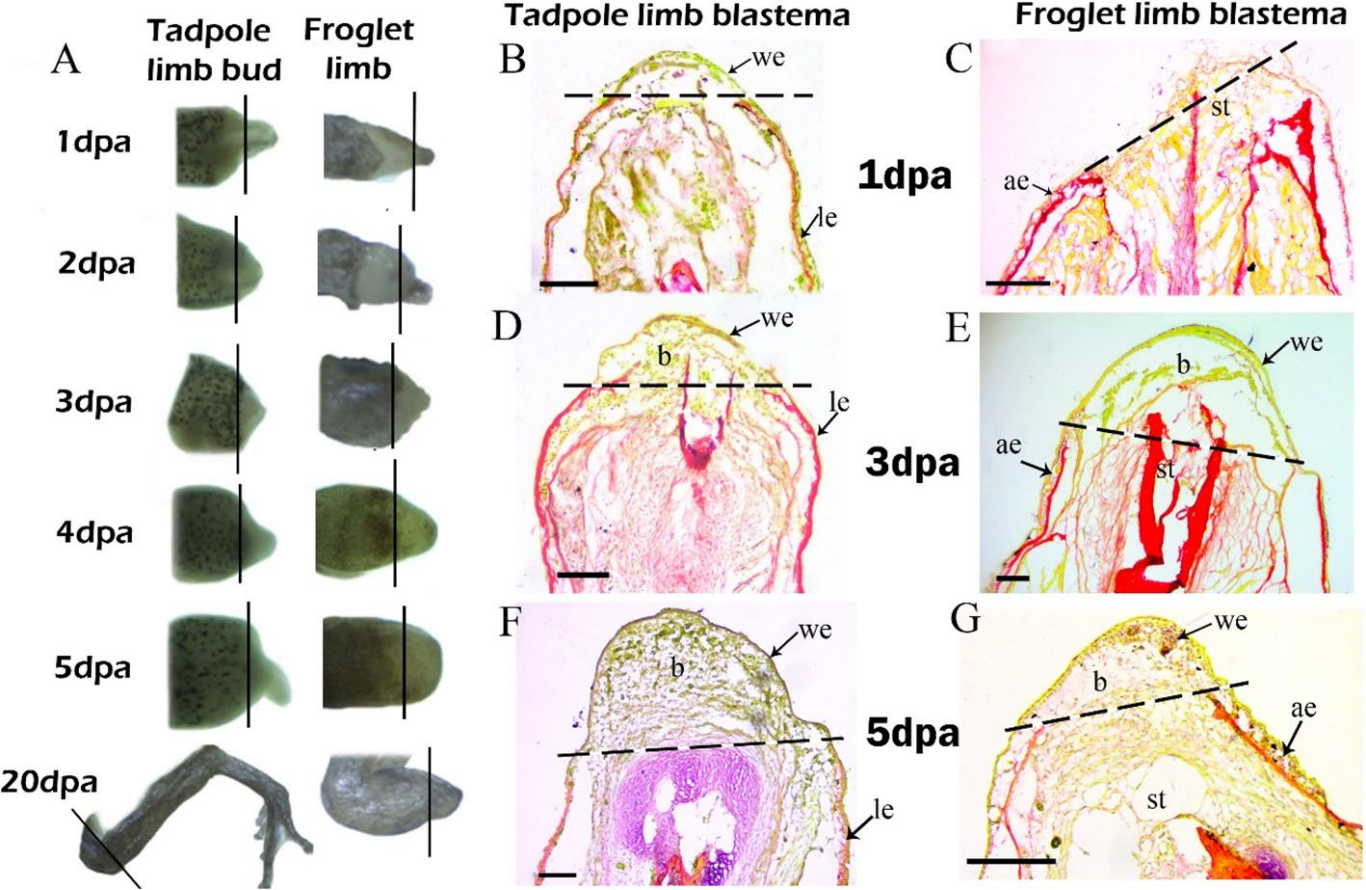
Early wound closure and accumulation of progenitor cells are hallmarks of successful limb regeneration in tadpoles of *P. maculatus*. **A** Comparative morphology of post amputated limb blastemas of tadpole and froglet (Not in scale); Black lines indicate plane of amputation; **B**: 1 dpa tadpole limb; **C** 1 dpa froglet limb; **D** 3 dpa tadpole limb; **E** 3 dpa froglet limb; **F** 5 dpa tadpole limb; **G** 5 dpa froglet limb. we- wound epithelium, b-blastema, le- larval epithelium, ae- adult epithelium, st-limb stump. Bars B-G = 100 μm, Dashed lines in **B**-**G** showing plane of amputation

Stage 56 tadpoles of *P. maculatus* regenerated their limbs post amputation forming a fully functional limb 20 dpa (Fig. 1A). Beyond stage 56 up to the onset of metamorphosis (stage 59), limb amputations resulted in wound healing and occasionally formed hypomorphic limbs (SFig. 2). The froglets, post-amputation did not regenerate their hindlimbs. The wound epithelium formed 6hpa in stage 56 tadpoles (SFig. 3A). The dermal progenitor cells seemed to migrate from the unamputated part of the limb towards the multi-layered wound epithelium at 1 dpa (Fig. 1B, SFig. 3B) and accumulated in large numbers 3 dpa to form a blastema (Fig. 1D, SFig. 3C). The wound epithelium appeared to be single-layered with a considerable accumulation of blastemal cells at 5 dpa (Fig. 1F, SFig. 3D). The blastema grew beyond the plane of amputation and started differentiating into digits 10 dpa (SFig. 3E-F). The wound epithelium was not visible at 1 dpa froglet limb (Fig. 1C). The wound epithelium formed 2 dpa (figure not shown) and became multi-layered 3 dpa with the accumulation of few mesenchymal cells (Fig. 1E). The froglet limb 5 dpa consisted of a multi-layered wound epithelium encompassing few mesenchymal cells interspersed with connective tissue (Fig. 1G).

### RNA-seq analysis

A *de-novo* reference transcriptome was generated from froglet hind limbs (Group 1), 3 dpa hind limb blastemas of froglets (Group 2), tadpole hind limbs (Group 3) and 3 dpa hind limb blastemas of tadpoles (Group 4). A summary of high-quality PE reads from the 12 samples (3 biological replicates for each group) is in STable 1 and the summary of transcripts, unigenes and coding sequences (CDS) is in STable 2. Out of the 28,427 CDS identified, 7493 CDS were without any blast hit and were novel and unique to *Polypedates maculatus*. Most of the blast hits were against *Nanorana parkeri* (SFig. 4). Group-wise CDS statistics are summarized in STable 3.

### GO annotation and KEGG pathway analyses

Gene ontology (GO) analyses showed that Molecular function had the highest number of CDS for all sample groups (STable 4). 12–13% of coding sequences annotated into GO categories and the rest of the genes were either novel i.e., without blast hits or not assigned into GO category. The pie charts and WEGO plots for GO categories are in SFigs.5–8. The CDS annotated in different GO categories were highest for 3 dpa tadpole blastema and lowest for intact froglet hindlimb. The WEGO plots identified transcripts for antioxidant activity and detoxification in the 3 dpa froglet blastemas but not for other sample groups. Quite a few genes for the immune system process were higher in 3 dpa tadpole and froglet blastemas relative their intact limbs. The source data of different GO categories of the four sample groups are in Supplementary file 1.

KEGG analysis of the predicted CDS was categorized into 29 KEGG pathways under five main categories: Metabolism, Genetic Information Processing, Environmental Information processing, Cellular processes, and Organismal systems. Out of the total annotated CDS, only 40–42% were KEGG annotated (STable 5). The largest cluster of genes belonged to signal transduction followed by transport and catabolism, the immune system, and the endocrine system for all four groups. The number of genes associated with development and regeneration was highest for 3 dpa tadpole limb blastema. The summary of KEGG pathway annotation is in STable 6. The source data of KEGG pathway analysis of the four sample groups is in Supplementary file 2. Transcripts of several signalling pathways such as Notch, Hedgehog, MAPK, cGMP-PKG, mTOR, Ras, TGFβ, Hippo, Wnt, etc. were detected in all sample groups but few regeneration-related transcripts were found to be exclusively expressed in the Group 4 (i.e., tadpole blastema) e.g., SHH, GLI3 of Hedgehog, AXIN2 and FZD6 of Wnt, DLL and NOTCH1 of Notch, FBN1, SMAD7 and NEO1 of TGFβ, LIMD1 and TEAD of Hippo signalling etc.

### Differential gene expression analysis

Differential gene expression analyses were done for all the four combinations of samples such as Combination 1 [Group 1 (intact hind limbs of froglets) and Group 3 (intact hind limbs of tadpoles)]; Combination 2 [Group 1 (intact hind limbs of froglets) and Group 2 (3 dpa blastemas of froglets)], Combination 3 [Group 3 (intact hind limbs of tadpoles) and Group 4 (3 dpa blastemas of tadpoles)] and Combination 4 [Group 2 (3 dpa blastemas of froglets) and Group 4 (3 dpa blastemas of tadpoles)]. The statistics of differentially expressed genes of the four combinations are in STable 7 and SFig. 9. DGE revealed the up-regulated and downregulated genes in the four combinations. Some of the differentially expressed genes of the four combinations were validated with quantitative real-time PCR (Supplementary file 3). Pearson’s correlation coefficient was positive showing a significant correlation between RNA-seq and RT-qPCR data (SFig. 10). The upregulated and downregulated genes of the four combinations are in Supplementary file 4.

#### A growing limb of tadpole vis a vis a completely formed froglet limb

**Fig. 2:**
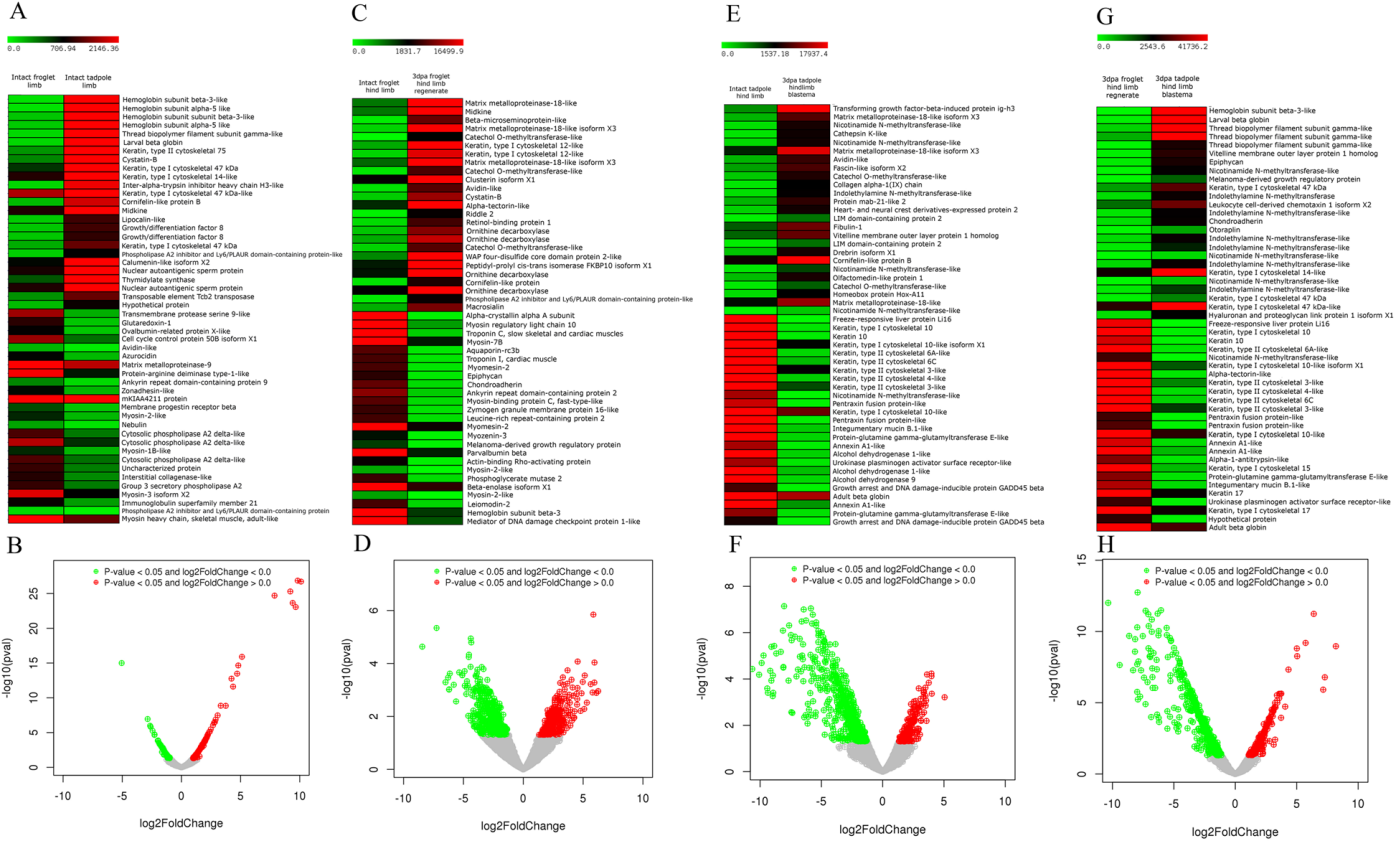
Differential gene expression analyses. **A** Heat map and **B** Volcano plot of normalized transcript levels between intact froglet and tadpole hindlimbs. **C** Heat map and **D** Volcano plot of normalized transcript levels between intact froglet hindlimb and 3 dpa froglet hindlimb blastema; **E** Heat map and **F** Volcano plot of normalized transcript levels between intact tadpole hindlimb and 3 dpa tadpole hindlimb blastema; **G** Heat map and **H** Volcano plot of normalized transcript levels between 3 dpa froglet and tadpole hindlimb blastemas

The intrinsic property of a homeostatic limb can be a determining factor for its regenerative ability. Differential gene expression analysis of the homeostatic tadpole and froglet limbs (Combination 1) identified the differences in their molecular repertoire. Four hundred ninety-four genes upregulated and 214 genes downregulated in the tadpole limbs relative to froglet limbs (STable 7, Fig. 2B).

The tadpole limb at stage 56 is still growing and differentiating. A higher expression of larval-specific haemoglobin (haemoglobin subunit alpha-5, haemoglobin subunit beta 3, larval beta globin), intermediate filament i.e., keratins (Keratin, type II cytoskeletal 75, Keratin, type I cytoskeletal 47 kDa, keratin, type I cytoskeletal 14) and thread biopolymer filament subunit gamma-like (TBFSG), cysteine and serine protease inhibitor (cystatin-B, inter-alpha-trypsin inhibitor) with log 2-fold changes> 4 found in tadpole limbs relative to froglet limbs (Fig. 2A). Genes related to cell cycle, DNA replication and repair, transcription and translation were also upregulated in the tadpole limb. The upregulated and downregulated genes of Combination 1 according to GO/KEGG/Function are in Supplementary file 4. Contrary, the froglet limb is completely differentiated and showed an increased expression of serine proteases (transmembrane protease serine 9, Serine protease 33), collagenases (matrix metalloproteinase 9, interstitial collagenase-like), inflammatory genes (Ras-related protein Rab-12, cytosolic phospholipase A2 delta, interleukin-8-like, azurocidin) and antioxidant (glutaredoxin) relative to tadpole limbs. A transcript was with log 2-fold change > 5 but without any blast hit i.e., unique to the species.

#### DGE analyses of 3 dpa froglet and tadpole blastemas relative to their respective homeostatic limbs showed upregulation of MMP’s and inflammatory genes

Differential gene expression analysis identified 329 upregulated and 505 downregulated transcripts in the 3 dpa blastema relative to the homeostatic froglet limb (STable 7). The heat map and volcano plots of the gene expression analyses are in Fig. 2C-D. Highly upregulated genes included genes responsible for ECM degradation (matrix metalloproteinase 18-like (MMP18-like) and its isoform X3, MMP-19), inflammation (midkine (MDK), phospholipase A2 inhibitor, ornithine decarboxylase, Ly6/PLAUR domain-containing protein-like, clusterin isoform X1,WAP four-disulfide core domain protein 2-like, avidin-like), tumour suppressors (β-microseminoprotein-like, catechol-O-methyltransferase), protease inhibitors (cystatin B), and keratinocytes (keratin type1 cytoskeletal-12-like, cornifelin-like protein B) etc. Additional upregulated genes were related to immune response and inflammation, serine proteases, cell cycle etc. (Combination 2 Upregulated; Supplementary File 4). The major downregulated genes were those related to myofibrillogenesis (myosin regulatory light chain 10, myosin-7B, myosin-2-like, myosin-binding protein C, fast-type-like, myomesin-2, myozenin-3, parvalbumin beta, ankyrin repeat domain-containing protein 2), calcium signalling (troponin C, troponin I), alcohol dehydrogenase activity, carbohydrate metabolism and proteoglycans (epiphycan (EPYC), chondroadherin (CHAD) and hyaluronan and proteoglycan link protein 1 (HAPLN1)) (Combination 2 Downregulated; Supplementary File 4).

Similarly, for Combination 3, DGE analysis revealed 213 upregulated and 678 downregulated genes in the tadpole limb blastema relative to the tadpole limb (STable 7; Fig. 2F). Highly upregulated genes in the tadpole blastema were those for ECM degradation (MMP18-like and its isoform X3, interstitial collagenase-like) and pro-inflammatory TGFβ induced protein ig-h3 (TGFβigh3) (Fig. 2E). Other remarkable upregulated genes included methyltransferases (nicotinamide n-methyltransferase (NNMT), and indole-ethylamine n-methyltransferase (INMT)), immune response, inflammation, and cell cycle (Combination 3 Upregulated; Supplementary File 4). The downregulated genes were freeze-responsive liver protein li16 (FRLP), cytoskeletal keratins, muscle contraction and carbohydrate metabolism. However, certain transcripts related to inflammation (pentraxin fusion protein, integumentary mucin B, annexin A1, urokinase plasminogen activator surface receptor-like) were also downregulated (Combination 3 Downregulated; Supplementary File 4) suggesting differential involvement of inflammation-related transcripts during limb regeneration.

#### Recipe for successful limb regeneration

The DGE analyses of the Combination 2 and 3 showed a similar gene expression profile for several genes indicating that differential regulation of those genes followed a similar path in both regenerating and non-regenerating blastemas. To understand the actual contribution of these differentially regulated genes in a regenerative response it was essential to carry out the DGE analyses between the 3 dpa tadpole blastemas relative to 3 dpa froglet blastemas (Combination 4). DGE analyses of Combination 4 showed 294 upregulated and 698 downregulated transcripts in the 3 dpa tadpole blastemas relative to the same day froglet blastemas (STable 7). DGE analyses between the blastemas unravelled several genes crucial for a regenerative response, elusive in the DGE analyses between homeostatic limbs and 3 dpa blastemas and hence gave a better clarity of the regeneration scenario (Fig. 2G-H). For example, some of the highly upregulated genes in the 3 dpa tadpole blastema like proteoglycans (EPYC, CHAD and HAPLN1), Keratin, type I cytoskeletal 47 kDa, MMP23-like, tumour suppressor (melanoma derived growth regulatory protein*,* otoraplin) and pattern formation (HOXA9, HOXC9) were not apparent in the DGE analyses of Combination 3. Similarly, downregulated genes like cytoskeletal keratins (Keratin, type I cytoskeletal 15, keratin, type I cytoskeletal 17 and keratin 17), ECM protein (α-tectorin like) and amphiregulin were also identified by comparing regenerating and non-regenerating blastemas. DGE analyses also revealed upregulation (as seen in Combination 3) of methyltransferases, ECM proteins and Wnt signalling and downregulation of inflammation, MMPs, apoptosis, oxidoreductase activity, cell adhesion, and keratinization (Combination 4; Supplementary file 4) in the tadpole blastemas.

#### Temporal expression of selected genes by qPCR analyses

**Fig. 3:**
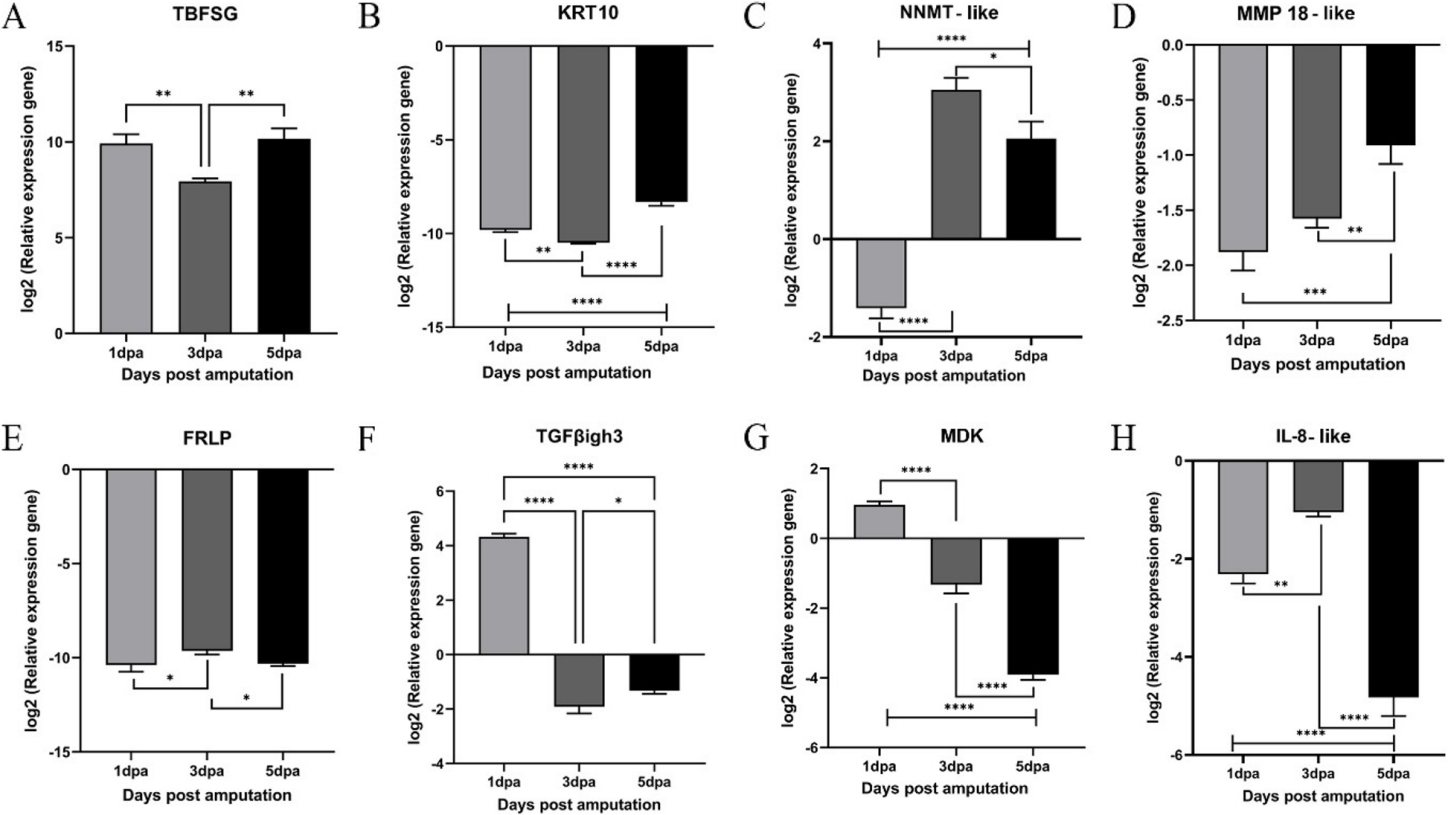
Differential expression patterns of transcripts by qPCR at different days post amputation tadpole blastemas relative to froglet blastemas. log2 values of normalized expression of TBFSG (**A**), KRT10 (**B**), NNMT-like (**C**), MMP-18like (**D**), FRLP (**E**), TGFβigh3 (**F**), MDK (**G**), IL-8 like (**H**) of 1,3 and 5 dpa tadpole blastemas relative to same day froglet blastemas. Asterisks indicate statistical significance (*: *p* < 0.05; **: *p* < 0.01; ***: *p* < 0.001; ****: *p* < 0.0001) Data are the mean of three biological replicates, each one with three technical replicates

The qPCR analyses of Combination 2 and 3 transcripts (i.e., tadpole and froglet blastemas relative to their respective homeostatic limbs) showed upregulation of TBFSG, MMP-18-like, FRLP, TGFβigh3, MDK, IL-8-like and LEF1 different days post-amputation (SFigs.11–12). KRT10, NNMT-like, EPYC (except 1 dpa) and INMT were upregulated in the tadpole blastemas (S Fig. 11) while they were downregulated in the froglet blastemas except for KRT10 at 5 dpa (S Fig. 12). The same DEGs from combination 4 were selected for comparative expression analysis at different time points post-amputation (Fig. 3). TBFSG, an intermediate filament protein, upregulated in the tadpole limb blastemas relative to that of the froglet (Fig. 3A). However, expression of KRT10 (Fig. 3B), MMP18-like (Fig. 3D), FRLP (Fig. 3E) and Interleukin 8-like (IL8-like) (Fig. 3H) downregulated in the regenerating blastemas. NNMT (Fig. 3C) showed the highest expression at 3 dpa while MDK (Fig. 3G) and TGFβigh3 expression was high only at 1 dpa and downregulated 3 and 5dpa (Fig. 3F).

### The regenerating tadpole limb blastema had a discrete composition of the extracellular matrix and the GAG+ myeloid cells contributed to the pro-regenerative extracellular matrix

**Fig. 4:**
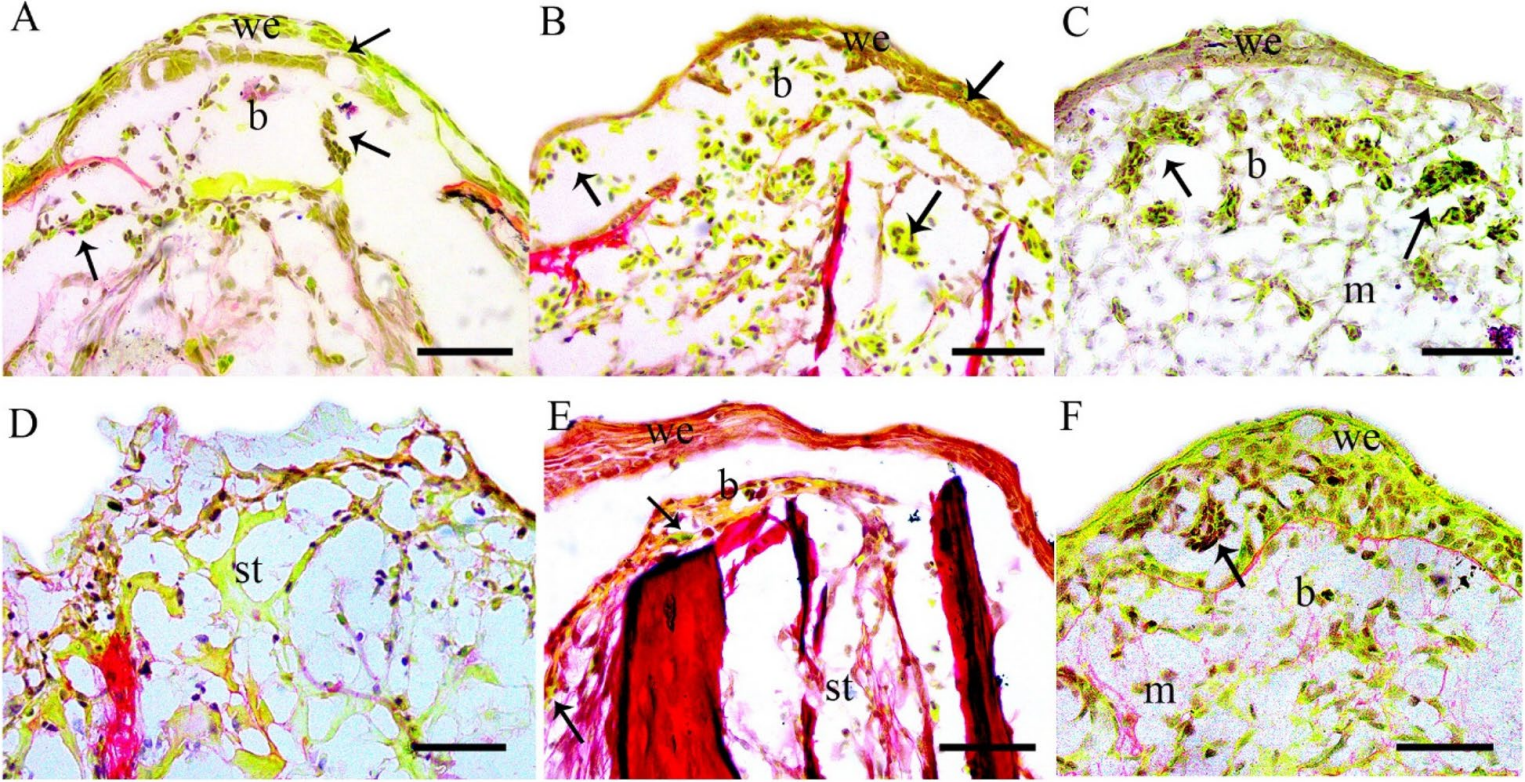
Glycosaminoglycan positive (GAG+) cells in tadpole and froglet hindlimb blastemas. **A** 1 dpa tadpole blastema; **B** 3 dpa tadpole blastema; **C** 5 dpa tadpole blastema showing formation of extracellular matrix (m) in the blastema; **D** 1 dpa froglet blastema showing absence of wound epithelium and blastema; **E** 3 dpa froglet blastema showing an adult type epidermis underlined by basement membrane enclosing the wound area.; **F** 5 dpa froglet blastema; Bars in A-C = 50 μm, D-F = 25 μm we- wound epithelium; b- blastema, st- limb stump, bm- basement membrane, m- extracellular matrix. Black arrows showing GAG+ cells

Pentachrome staining (that stains collagen red and glycosaminoglycans purple) of the tadpole blastemas showed a high accumulation of glycosaminoglycan positive (GAG+) cells (Fig. 4A-C). Their number gradually declined as the blastema grew and differentiated (SFigs.3E-F). The tadpole blastema at 5 dpa had a non-collagenous matrix that appeared as a network of glycosaminoglycans (light violet in colour) (Fig. 4C). The number of GAG+ cells was however meagre in the froglet blastemas. The few cells that accumulated below the wound epithelium in the 1 dpa and 3 dpa froglet blastemas were not GAG+ (Fig. 4D-E). The froglet blastema at 5 dpa had a collagenous matrix (reddish colour network) and very few GAG+ cells in the wound epidermis (Fig. 4F).

The differences in extracellular matrix composition were also evident from transcriptome data. The DEGs of various extracellular matrix proteins like collagens, proteoglycans, glycosaminoglycan synthesis, glycoproteins and ECM organization of Combination 4 (i.e., 3 dpa limb blastemas of tadpole and froglet) are in Supplementary file 5. As stated above, proteoglycans EPYC, CHAD and HAPLN1 were upregulated in the tadpole limb blastema while there was no significant difference in the expression of other proteoglycans. The log2 fold change in expression of collagens COL2A1, COL9A1, COL9A2, and COL9A3 was more than two times in the tadpole blastema relative to froglet blastema. Other collagens upregulated in the tadpole blastema were COL5A1, COL6A1 AND COL11A1 while COL10A1 upregulated in the froglet blastema. Various enzymes necessary for glycosaminoglycan synthesis were not significantly different during regeneration and healing.

**Fig. 5:**
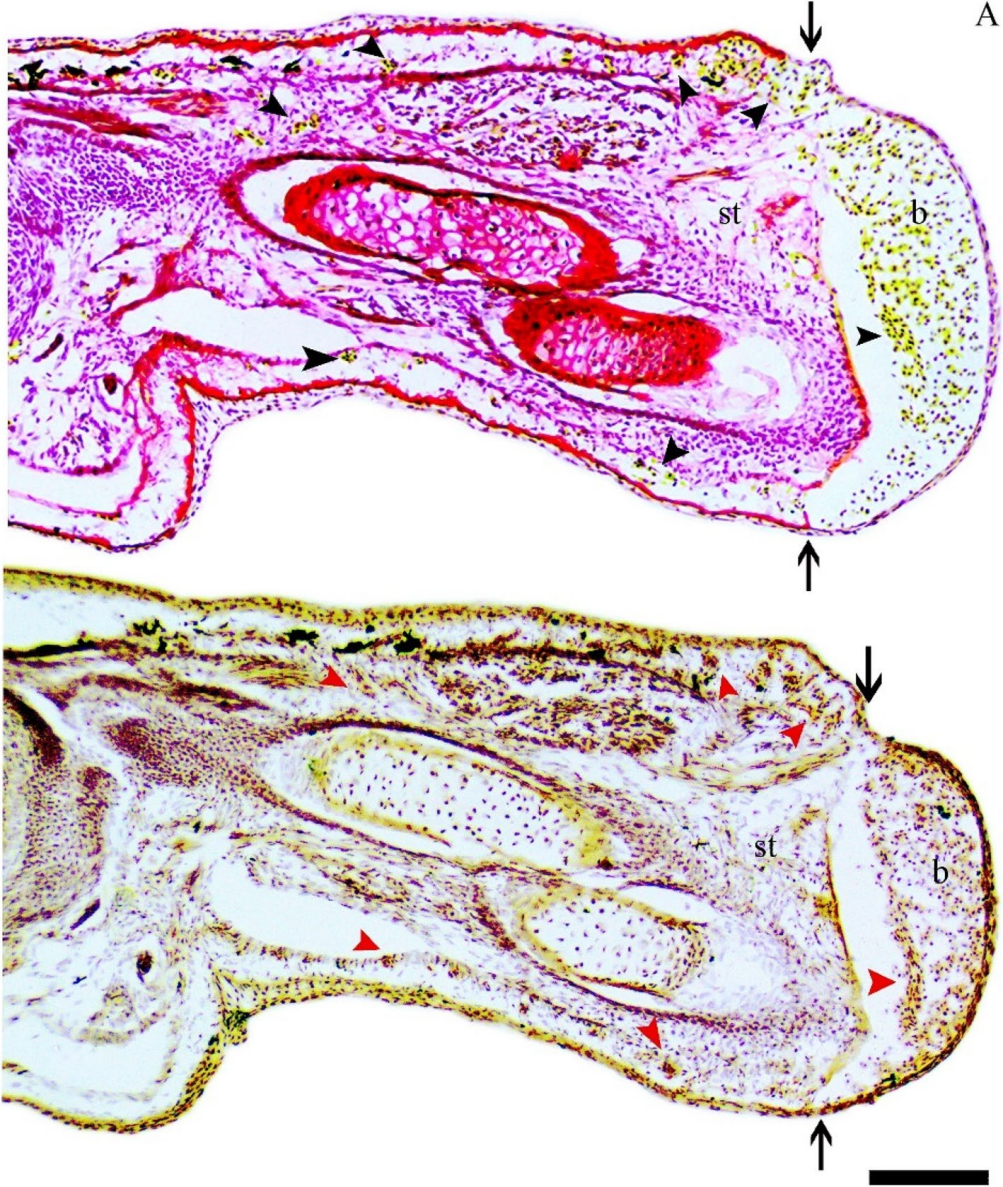
Longitudinal sections through tadpole limb with 1 dpa blastema showing dermal GAG+ cells to be NAE+. **A** Pentachrome stained section showing GAG+ cells (Black arrowheads), **B** NAE stained serial section of (**A**) showing myeloid cells (NAE+) (Red arrowheads). Black arrows show the plane of amputation. Bars = 125 μm. b- blastema, st-stump

Naphthyl acetate esterase staining revealed a high density of myeloid cells in the tadpole blastemas that were positive for glycosaminoglycans (GAG+). Such cells were found in the dermal regions of the unamputated part of the tadpole limbs and also accumulated in the blastema (Fig. 5A-B). The myeloid cells (NAE+) occupied a significant portion of the blastema area 1 dpa, and their numbers gradually declined at 5 dpa (SFigs.14A-C, G). Few myeloid cells were found beneath the wound epithelium at 3 and 5 dpa froglet blastemas (SFigs.14D-F, H). The GAG+ myeloid cells (SFigs.13A-B) appeared to arise from the degenerating muscle fibres rather than the dermis (SFig. 14E). The myeloid cells were a subset of GAG+ cells in both tadpole and froglet blastemas as their numbers were lower than GAG+ cells (STable 8). Notably, the number of both GAG+ and NAE+ cells declined in the tadpole blastema over time while in a froglet blastema a comparable number of myeloid cells were sustained (SFigs.14G-H).

### A species-specific expression of EPYC, INMT, LEF1 and regeneration exclusive expression of SALL4 in the tadpole blastemas

**Fig. 6:**
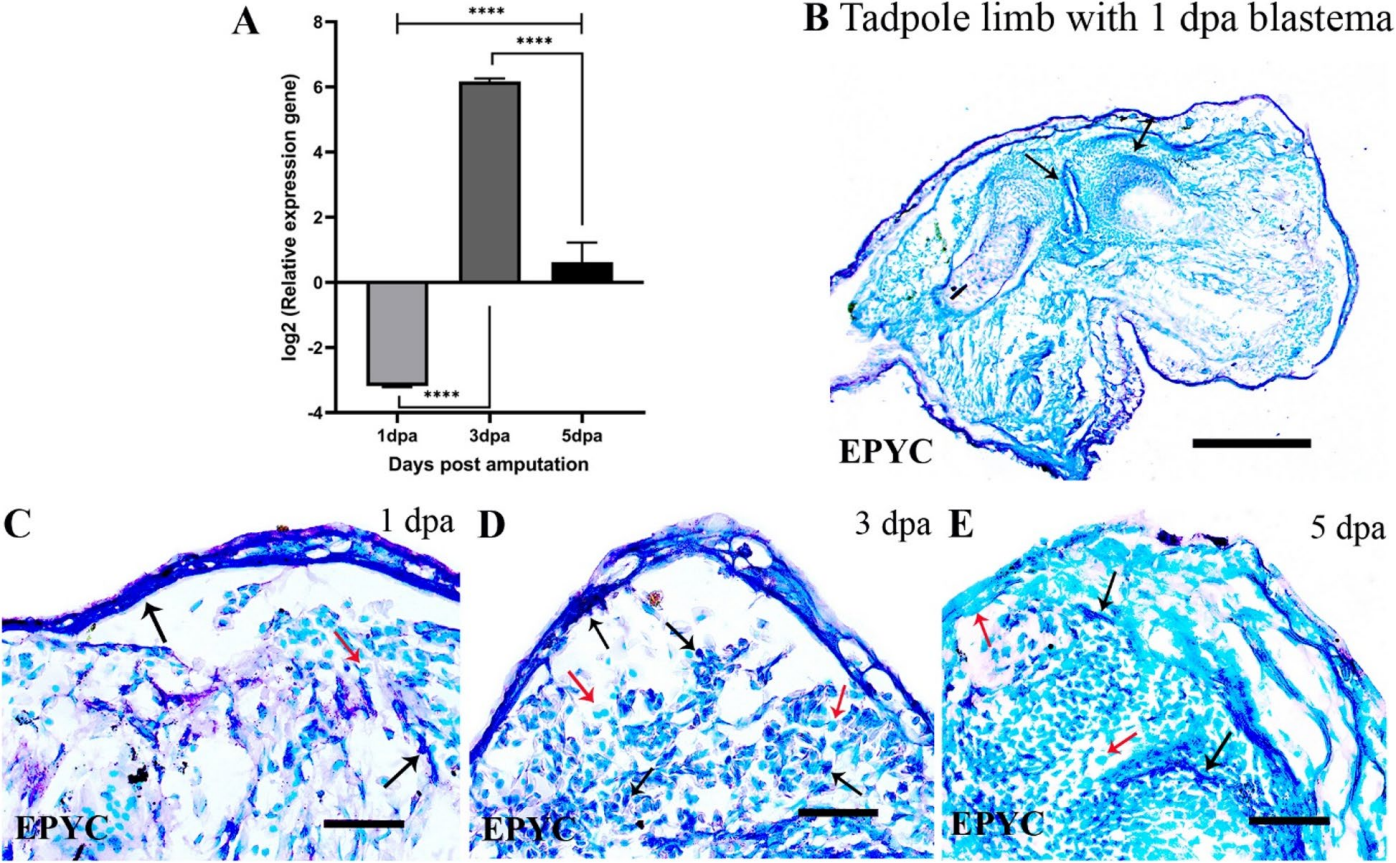
Expression of epiphycan (EPYC) during tadpole limb regeneration. **A** log2 values of normalized expression of EPYC in the 1,3 and 5 dpa tadpole blastemas relative to the same day froglet blastemas. **B**-**E** RNA in situ hybridization of EPYC in the tadpole hindlimb and blastemas. Bars in B = 250 μm; C-E, =50 μm. Black arrows indicate positive hybridization and red arrows indicate no hybridization

EPYC, a proteoglycan, was upregulated in the 3 and 5 dpa while it was low in the 1 dpa tadpole blastemas relative to the same-day froglet blastemas (Fig. 6A). in situ hybridisation of EPYC in the tadpole blastemas revealed its expression in the wound epithelium 1 dpa (Fig. 6C), wound epithelium and blastemal mesenchymal cells 3 dpa (Fig. 6D) and a few places in the wound epithelium and mesenchymal cells 5 dpa (Fig. 6E). EPYC was also expressed at the junction of bones (Fig. 6B), dermal myeloid cells but not in the bone marrow cells of the unamputated portion of the tadpole limbs (SFigs.15A-B, D).

**Fig. 7:**
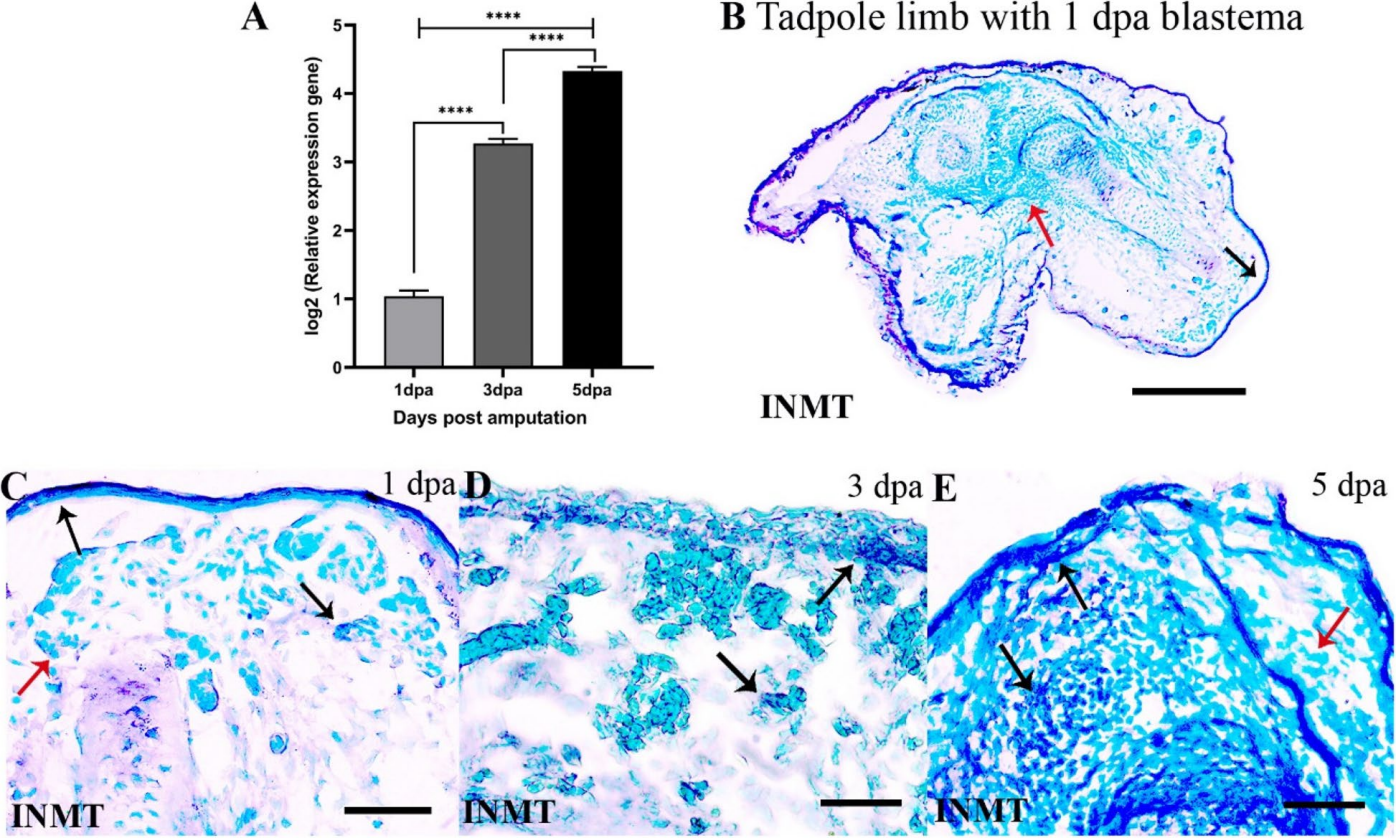
Expression of indole ethylamine n methyl transferase (INMT) during tadpole limb regeneration. **A** log2 values of normalized expression of INMT in the 1,3 and 5 dpa tadpole blastemas relative to the same day froglet blastemas. **B**-**E** RNA in situ hybridization of EPYC in the tadpole hindlimb and blastemas. Bars in B = 250 μm; C-E = 50 μm. Black arrows indicate positive hybridization and red arrows indicate no hybridization

INMT*,* a methyltransferase, was highly upregulated in the tadpole blastemas compared to same day froglet blastemas (Fig. 7A) and its expression increased with the growth of the tadpole blastema. It localised to the wound epithelium and mesenchymal cells of the tadpole blastemas (Fig. 7C-E) and in the unamputated limb, it was found in the dermal myeloid cells, bone marrow cells and extracellular matrix around the bone (Fig. 7B; SFigs.15A-C).

**Fig. 8:**
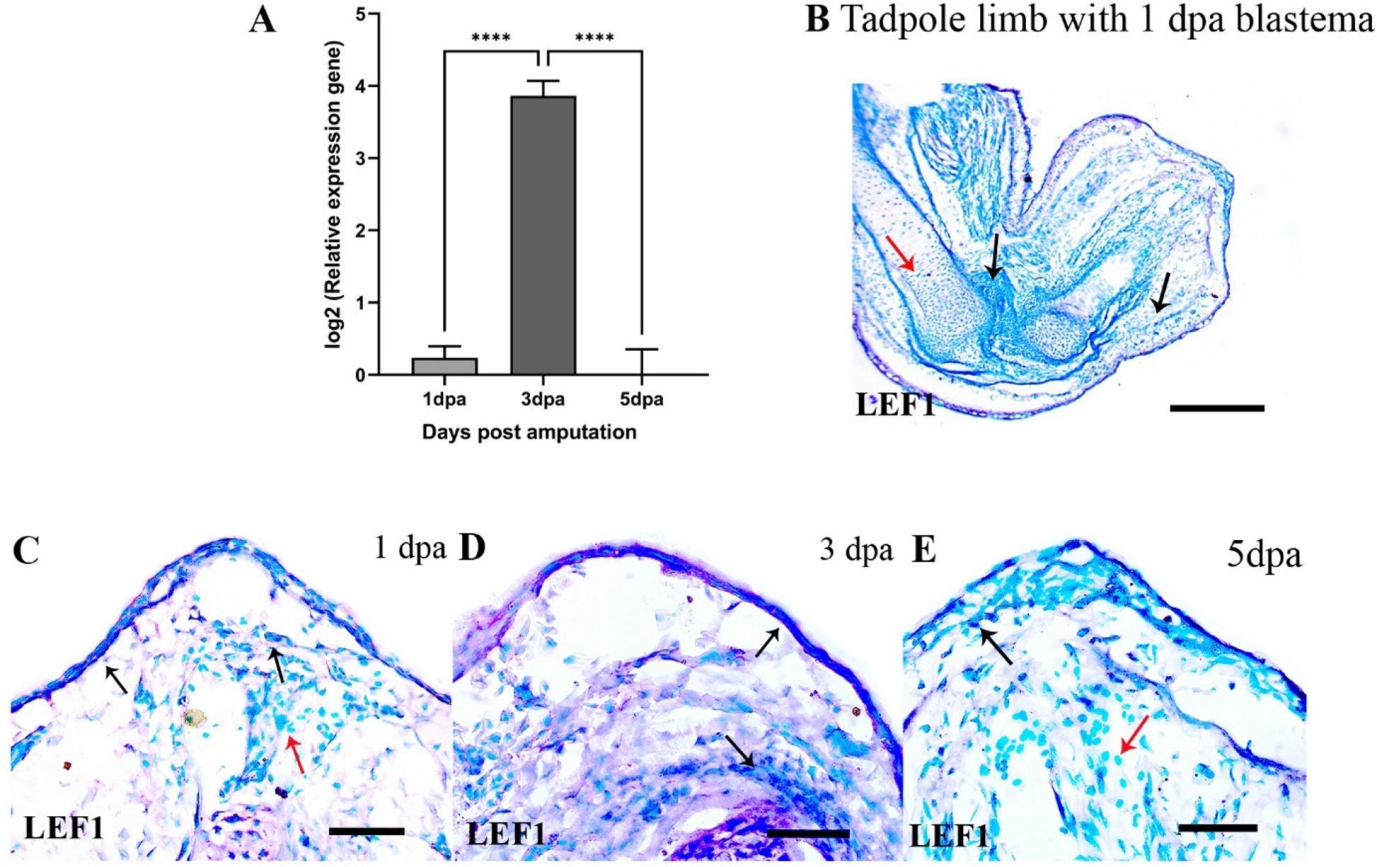
Expression of lymphoid enhancer binding factor 1(LEF1) during tadpole limb regeneration. **A** log2 values of normalized expression of LEF1 in the 1,3 and 5 dpa tadpole blastemas relative to the same day froglet blastemas. **B**-**E** RNA in situ hybridization of LEF1in the tadpole hindlimb and blastemas. Bars in C = 250 μm; D-F = 50 μm. Black arrows indicate positive hybridization and red arrows indicate no hybridization

LEF1 expression was highest at 3 dpa and decreased at 5 dpa tadpole blastemas (Fig. 8A), relative to the same day froglet blastemas. in situ hybridisation of LEF1 was confined to the blastemal mesenchymal cells and the basal epithelial cells of the wound epithelium at 1 and 3 dpa (Fig. 8B-D) and few blastemal mesenchymal cells at 5 dpa (Fig. 8E). LEF1 expression was also detected in the unamputated portions of the tadpole limb in the dermal myeloid cells, in the degrading myofibres and the extracellular matrix near the bone (Fig. 8B, SFigs.15A-B, F).

**Fig. 9:**
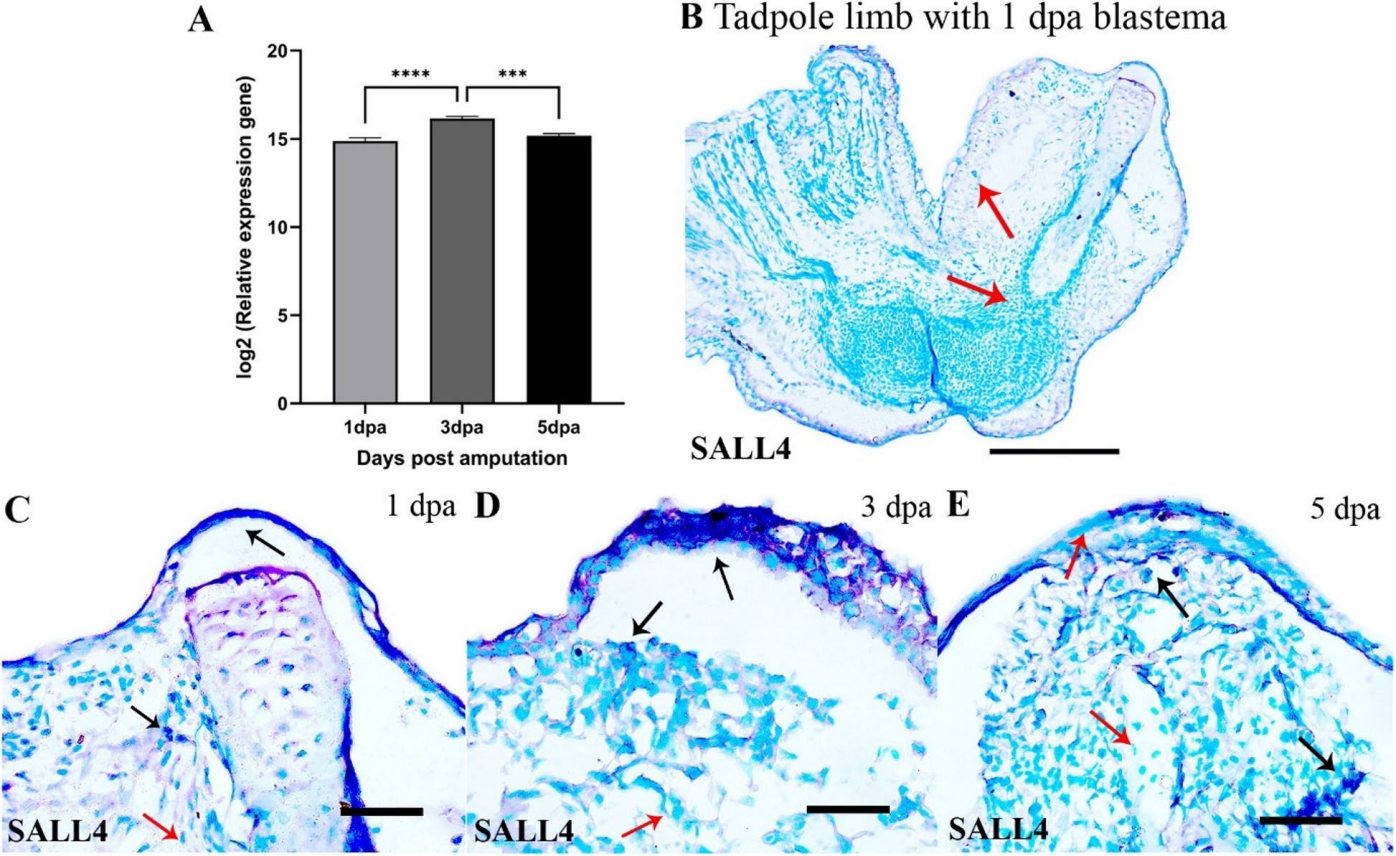
Expression of Sal-like 4 (SALL4) during tadpole limb regeneration. **A** log2 values of normalized expression of SALL4 in the 1,3 and 5 dpa tadpole blastemas relative to the homeostatic tadpole limb. **B**-**E** RNA in situ hybridization of SALL4 in the tadpole 1,3 and 5 dpa blastemas. Bars in B = 250 μm; C-E = 50 μm. Black arrows indicate mRNA hybridisation. Red arrows indicate no hybridisation

The expression of SALL4 was highest at 3 dpa that declined subsequently in the tadpole blastemas (Fig. 9A) but its expression could not be detected in the froglet blastemas (Fig. 9B). in situ hybridisation in the tadpole blastemas showed positive signals for SALL4 in the wound epithelium and blastemal mesenchymal cells under the wound epithelium at 1 dpa and 3 dpa (Fig. 9C-D) and by 5 dpa there was decreased hybridisation in the wound epithelium (Fig. 9E). SALL4 also localised to the dermal myeloid cells in the unamputated limb portions (SFigs.15A-B, E).

## Discussion

Limb regeneration is a complex process and is not understood completely. This study presents the first de novo reference transcriptome of regenerating and non-regenerating limb blastemas of *P. maculatus*, a sub-tropical frog from India. The study describes conservative and species-specific regulators of limb regeneration and the inherent property of the limb as a determinant for successful limb regeneration. The GAG+, EPYC+, INMT+, LEF1+ and SALL4+ myeloid cells contributed to the pro-regenerative extracellular matrix during limb regeneration.

The limb regeneration ability in *P. maculatus* was unambiguously different from *Xenopus laevis*. The limbs of *X. laevis* tadpoles regenerate at NF stages 52–53, enter a refractory period at stages 56–57 and generate a spike post-amputation in the froglets [27, 28]. However, the limbs of *P. maculatus* were regeneration competent at NF stages 52–56, formed hypomorphic limbs at stages 57–58 and regeneration incompetent stage 59 onwards. Transcriptomics and DGE analysis revealed that the tissue microenvironment in the froglet limbs was mostly inflammatory which suppressed limb regeneration. Relatively higher expression of serine proteases, collagenases, and inflammatory genes along with reduced expression of cysteine and serine protease inhibitors in the homeostatic froglet limbs possibly created an environment that was capable of inducing inflammation and did not support regeneration. A prolonged inflammation is pro-healing rather than pro-regenerative [20, 29]. The serine proteases promote inflammatory protein expression and directly influence the degradation of the extracellular matrix, loss of epithelial barrier function and MMP-9 activation during ocular inflammation [30]. Inter alpha trypsin inhibitor inhibits serine proteases during sepsis [31] and imparts protease and hyaluronidase inhibitory activity in various pathological conditions [32]. However, noggin, described as a factor of regeneration incompetency in *Xenopus* [10] was not associated with the regeneration incompetency in the *P. maculatus* froglet limbs as there was no differential expression between froglet and tadpole limbs.

Several genes showed a similar expression profile e.g., MMP’s and inflammatory genes were upregulated but FRLP was downregulated in the froglet and the tadpole blastemas relative to their homeostatic limbs although tadpole blastemas regenerated and froglet blastemas healed. For achieving clarity on the expression of such genes during regeneration, expression was analysed in the tadpole blastemas relative to the froglet blastemas. Activation of the immune system and upregulation of matrix metalloproteinases is a common response to limb amputation in amphibians causing histolysis of extracellular matrix in the wound area [6, 15, 33]. However, DGE analyses of tadpole blastemas relative to froglet blastemas revealed that the MMPs (interstitial collagenase, MMP9, MMP13-like, MMP18-like) and immune response and inflammatory genes were majorly upregulated in the froglet blastemas causing healing.

Real-time PCR analysis showed downregulation of IL8-like in the tadpole blastemas relative to froglet blastemas affirming a reduced inflammation as a cause for regeneration. However, IL-8 promotes limb regeneration in axolotls by recruiting myeloid cells to the wound area [8]. Although myeloid cells were recruited to the wound area and promoted limb regeneration as discussed below, the present study could not establish any link between IL8-like and myeloid cells. MDK seems to be the cytokine that recruits myeloid cells as evident from its upregulation at 1dpa tadpole blastemas. However, it is downregulated from 3dpa onwards indicating resolution of inflammation. MDK has also been reported to resolve inflammation during axolotl limb regeneration [34]. TGFβ induced (igh3) known to promote wound healing in vitro [35] has the same expression pattern as MDK and seems to have similar functions to MDK.

Contrary, successful regeneration in tadpoles involved the upregulation of blood-forming and pattern-formation transcripts, proteoglycans, methyltransferases, tumour suppressors and downregulation of FRLP, inflammation, MMP and keratins. FRLPs are expressed in response to ischemia [36] and their increased expression in the froglet blastema probably shuts down the initiation of major signalling pathways. Following limb amputation, the wound area becomes anoxic without any blood vessels. But the increased expression of haemoglobin and larval beta-globin transcripts in the tadpole blastema indicates vascularization of the wound area causing downregulation of the FRLPs to bring about successful regeneration. Keratins are known to support regeneration in *Xenopus* [37] and axolotl [38], but in the present study, most of the keratins were downregulated in the tadpole blastemas except keratin type I cytoskeletal 47 kDa. Amphiregulin described as a regeneration agonist in axolotl [19] was also downregulated in the *P. maculatus* tadpole blastemas. Several signalling pathways described in other amphibian regeneration models [8, 14, 39, 40] were identified in the homeostatic limbs and blastemas of tadpoles and froglets but few molecules of Hedgehog, Notch, Hippo, TGFβ, Wnt etc. were found to be exclusively expressed in the tadpole blastemas. Limb patterning gene SHH of the Hedgehog signalling pathway was not expressed in the *P. maculatus* froglet blastemas as reported earlier in *Xenopus* [23].

A regeneration-permissive ECM is essential during regeneration [41–43] and myeloid lineage is essential to form a regeneration-permissive environment post-tail amputation in *Xenopus* [44]. In the present study, resident dermal myeloid cells were the reservoirs of glycosaminoglycans (GAG+) that perhaps migrated and accumulated in the tadpole blastemas. Dermal myeloid cells were missing in the froglet blastemas populated with GAG+ myeloid cells derived from the damaged tissues in the wound site. In the salamander limb blastema, dermal fibroblasts synthesise sulphated glycosaminoglycans and are the major contributors of blastemal cells [45], however, in the present study keratinocytes and myeloid cells in the tadpole blastemas contained sulphated glycosaminoglycans. Studies on murine wound healing have reported the myeloid origin of fibroblasts that contributed to healing wounds [46, 47]. The dermal myeloid cells of the present study may transform into fibroblasts and probably migrate to the wound area to secrete glycosaminoglycans. Additionally, the dermal myeloid cells can also be the M2-like reparative and anti-inflammatory myeloid cells as seen in the tail regeneration-competent *Xenopus* tadpoles [44]. Further studies using fibroblast-specific markers and M2-like myeloid markers can recognize such a possibility*.* Glycosaminoglycans in the keratinocytes indicated their additional role in creating a regenerative wound epithelium. Thus, the presence of a large number of GAG+ dermal myeloid cells accompanied by lower expression of serine proteases, MMPs, inflammatory genes and antioxidants made the tadpole limbs regeneration competent.

Glycosaminoglycans like heparan sulphate proteoglycans are associated with zebrafish fin regeneration [48], and axolotl limb regeneration [49, 50]. Hyaluronan and proteoglycan link protein 3 was upregulated in the regenerating epithelium of axolotl limbs [51] and chondroitin sulphate proteoglycans are necessary for axonal regeneration and growth [52]. We report here the expression of three proteoglycans epiphycan, chondroadherin and hyaluronan and proteoglycan link protein 1 in a regenerating limb blastema of an amphibian. Epiphycan, a dermatan sulphate proteoglycan, has been associated with epiphyseal cartilage during mouse embryonic development [53] but probably never with any regenerative program. Keratinocytes and macrophages express various proteoglycans during homeostatic and pathological conditions [54, 55] and in the present study, epiphycan expressed in keratinocytes and myeloid cells during limb regeneration. Dermatan sulfate (DS) is the most abundant glycosaminoglycan in human wound fluid and perhaps serves as a cofactor for FGF-10 to expedite healing by stimulating keratinocyte activity [56] and probably epiphycan performs a similar function during limb regeneration in *P. maculatus*. Cells having a stellate morphology in the dermis and loose connective tissues surrounding limb tissues synthesised heparan sulphate proteoglycans during limb regeneration in the axolotls [50]. However, the GAG+ dermal myeloid cells of *P. maculatus* were elliptical or round and we did not encounter any cells with stellate morphology that were GAG+. Our study thus proposes a discrete and species-specific distribution of sulphated glycosaminoglycans for successful limb regeneration in *P. maculatus*. Further studies on proteoglycans of this species are underway.

Another important component of the ECM-the collagens was differentially expressed and specific for *P. maculatus*. The 5dpa froglet blastema can be aptly called a fibroblastema due to the accumulation of collagen. Failure in regeneration in axolotl limbs has been attributed to the deposition of collagen 1 and 4 due to early macrophage depletion [42]. However, there was no significant difference in the expression of COL1 and 4 in the regenerating and non-regenerating blastemas of *P. maculatus*. A recent study identified the stem cell factor SALL4 as a critical regulator of scar-free wound healing in axolotl [57] as it regulates the expression of type I and type XII collagens [41]. In the present study, SALL4 expression was the highest 3dpa tadpole blastema and probably prevented the deposition of pro-healing collagen (in this case COL 10). Significantly, SALL4 expression was exclusively specific to the tadpole and localised to the wound epithelium and dermal myeloid cells of the regenerating blastema. However, no expression of SALL4 was recorded in the froglet limb and blastemas either by transcriptomics or real-time PCR analyses establishing it as a regeneration-specific gene. Apart from proteoglycans and collagens, several ECM proteins were upregulated in the tadpole blastema suggesting a discrete architecture of the ECM during regeneration relative to healing.

Upregulation of various isoforms of methyl transferases like INMT and NNMT in the tadpole blastema was another noticeable discovery of this study unreported in many amphibian regeneration models. INMT is a methyl transferase that methylates tryptamine and biogenic amines to N, N-dimethyltryptamine (DMT) endogenously in humans and several other mammalian tissues and occurs in several isoforms [58]. In mammals, DMT is a natural endogenous ligand of the σR-1 receptor associated with several cellular functions, including neurogenesis [59]. INMT was detected not only in the basal wound epithelium and mesenchymal cells of the tadpole blastema but also in the dermal myeloid cells in the unamputated tadpole limb that perhaps migrated to the wound area. NNMT is known to promote epithelial-mesenchymal transition in gastric cancer cells [60, 61] and is probably involved in similar processes in the tadpole blastema.

LEF1 is a co-transcription factor of the canonical Wnt signalling pathway and its expression in the adult fibroblasts regenerated adult mice skin in a scarless manner [62]. LEF1 expression in the wound epithelium and the dermal myeloid cells suggested activation of canonical Wnt signalling in the 3 dpa tadpole blastema that diminished at 5 dpa because of the upregulation of Wnt antagonists (dickkopf related protein 2, Wnt inhibitory factor 1) at 3 dpa. LEF1 is a marker of the regeneration organizing cells during *Xenopus* tail regeneration [63]. LEF1 in the dermal myeloid cells suggests a possibility of they being the regeneration organizing cells in *P. maculatus* limb regeneration. Gain of function and loss of function studies are warranted in this regard.

## Conclusions

The present study describes the limb regeneration mechanisms of a non-model amphibian *Polypedates maculatus* by comparative transcriptomics of regenerating and regeneration deficient limb blastemas at different life-history stages. The study discovers several conserved strategies of regeneration like creation of a wound epithelium and a pro-regenerative ECM and down regulation of inflammation to form a blastema. However, discrete and species-specific differences were recognised in the constituents of the ECM as well as transcripts that regulated inflammation and contributed to limb regeneration. Thus, this study gives useful insights into the variety of molecules that can be involved in creating a regeneration-permissive environment. Additional studies on other non-model amphibians can give more information on the different kinds of proteins involved during limb regeneration and provide more scope for developing alternate strategies to create a regeneration-permissive environment in regeneration- deficient animals, the main goal of regenerative medicine.

## Materials and methods

### Rearing procedures of animals and limb amputations

Foam nests of *Polypedates maculatus* were collected from undisturbed areas near Baripada, Odisha, India from July–September. The foam nests were kept in laboratory conditions in glass aquariums simulated for amphibious conditions. The average temperature and humidity during that period remained at 30 °C and 81% respectively. The tadpoles were staged according to Nieuwkoop and Faber [64]. The tadpoles hatched out of the foam nests at stage 43 and subsequently reared in dechlorinated water in glass tubs up to metamorphosis. The tadpoles started feeding at stage 46 and were fed with boiled *Amaranthus* greens and egg yolk during the rearing period. Upon reaching the metamorphic climax at stage 59, the metamorphosing tadpoles were again transferred back to amphibious conditions in the glass aquariums until metamorphosis. The metamorphosing tadpoles were not fed during this period. For hindlimb amputations, stage 56 tadpoles and metamorphosed froglets were immobilized with MS222 and ice slabs, respectively, and the hindlimbs were amputated at mid-zeugopod level with a sharp blade on a marble plate under sterilized conditions. After limb amputations, the tadpoles were released in dechlorinated water while the froglets were released into amphibious conditions.

### Pentachrome and NAE staining

Intact hind limbs and hind limb blastemas 1–10 days post-amputation (dpa) of regenerating (tadpole) and non-regenerating (froglet) limbs were fixed in 10% neutral buffered formalin and paraffin-embedded. 5 μ sections were cut using a rotary microtome and stained with a new pentachrome stain [65]. The serial sections of early tadpole and froglet blastemas (1–7 dpa) were taken for α-naphthyl acetate esterase (NAE) staining. Briefly, the slides were fixed in Citrate-Acetone-Methanol Fixative for 1 minute at room temperature and stained with α-Naphthyl Acetate Solution for 30 minutes at 37 °C. Slides were counterstained with Mayer’s hematoxylin. Images of sections were imported to ImageJ software and converted to 8-bit binary images, and particle counting was used to quantify the positive staining of cells. Data were then exported for further analysis. Variations were analysed by one-way ANOVA followed by Tukey’s HSD test in tadpoles and by Student’s t-test in froglets. Significant differences were revealed by *p* values < 0.05. All statistical analyses were performed, and graphs were prepared with GraphPad Prism 9.3.1.

### Sample collection for RNA-seq analysis

Samples for RNA-seq analysis were prepared by collecting pools of intact hind limbs and 3 days post-amputation (dpa) hind limb blastemas of tadpoles and froglets. Although froglets do not regenerate limbs, a bump was observed 3dpa and referred to as blastema, for convenience in the entire text. The samples were designated as Group 1 (intact hind limbs of froglets), Group 2 (3 dpa blastemas of froglets), Group 3 (intact hind limbs of tadpoles) and Group 4 (3 dpa blastemas of tadpoles). Three biological replicates were made for each group. Samples were collected and stored in RNA later solution and transported on dry ice to Eurofins Genomics Pvt. India Ltd., Bengaluru for RNA isolation and de novo RNA sequencing.

### RNA isolation and Illumina library preparation

Total RNA was isolated using a commercially available Quick-RNA Miniprep Plus kit (ZYMO Research) as per the manufacturer’s instructions. The qualities and quantities of the isolated RNA were checked on 1% denaturing RNA agarose gel and NanoDrop, respectively. The QC-passed RNA samples were processed for paired-end library preparation using TruSeq stranded mRNA Library Prep kit as per the kit protocol. The libraries were sequenced on the Illumina NextSeq500 platform using 2 × 75 bp chemistry.

### De-novo transcriptome assembly

The sequenced raw data for the 12 samples from the 4 groups (3 biological replicates of each group) were processed to obtain high-quality concordant reads using Trimmomatic v0.38 to remove adapters, ambiguous reads, and low-quality sequences (reads with more than 10% quality threshold (QV) < 20 Phred score). The resulting high-quality (QV > 20), paired-end reads were used for *the* de novo assembly of the samples. The high-quality reads of all 12 samples were pooled together and assembled into transcripts using Trinity de novo assembler (v2.8.4) with a kmer of 25. The assembled transcripts were further clustered using CD-HIT-EST-4.6 to remove isoforms during assembly and to define Unigenes. Unigenes having > 80% coverage at 5X read depth were considered for downstream analysis. Transdecoder (v5.3.0) was used to predict coding sequences from the above-mentioned unigenes. Functional annotation of the CDS was performed using the DIAMOND (BLASTX alignment mode) program, a BLAST-compatible local aligner for mapping translated DNA query sequences against a non-redundant protein database from NCBI. A few query sequences were also aligned in blastn. Sample group-wise CDS identification was done by pooling the reads group-wise (Groups 1, 2, 3 and 4) and mapped on the final set of pooled CDS using BWA (−mem) toolkit. The read count values were calculated from the resulting mapping and those CDS having at least 80% coverage and 5X read depth were considered for downstream analysis for each of the sample groups. The bioinformatics workflow is in SFig. 1.

### Gene ontology and KEGG pathway analysis

Gene Ontology (GO) analyses of the CDS identified for each of the 4 groups were carried out using the Blast2GO program. The GO mapping used BlastX result accession IDs to retrieve UniProt Ids making use of PIR including PSD, UniProt, SwissProt, TrEMBL, RefSeq, GenPept and PDB databases. To identify the potential involvement of the predicted CDS in biological pathways, the CDS of the 4 groups were mapped to reference canonical pathways in KEGG (frog). The output of the KEGG analysis included KEGG Orthology (KO) assignments and corresponding Enzyme Commission (EC) numbers and metabolic pathways of predicted CDS using the KEGG automated annotation server, KAAS [66].

### Differential gene expression analysis

Differential gene expression analysis between commonly expressed CDS of the 4 groups was done using the DESeq package in the following combinations:

- Combination 1: Groups 1 and 3
- Combination 2: Groups 1 and 2
- Combination 3: Groups 3 and 4
- Combination 4: Groups 2 and 4

An average linkage hierarchical cluster analysis was performed on the top 50 differentially expressed genes using multiple experiments viewer (MeVv4.9.0). The Eurofins proprietary R script was used to depict the graphical representation and distribution of differentially expressed genes along dimensions of biological as well as statistical significance (Volcano plots).

### Quantitative real time-PCR (qRT-PCR)

Total RNA was isolated from pooled tissues from intact hindlimbs and 1, 3, and 5 dpa hind limb blastemas of regenerating tadpoles and non-regenerating froglets using TRIzol Reagent (Life Technologies). The total RNA was purified with RNeasy mini kit (Qiagen) and reverse transcribed to single-stranded cDNA with iScript Adv cDNA kit (BioRad) following the manufacturer’s instructions. Expression levels of specific mRNA’s were determined by qPCR using gene-specific primer pairs (STable 9) using SSO Advanced Universal SYBR Green supermix kit (BioRad) following manufacturer’s instructions on a Biorad Real-Time PCR system. Melt-curve analysis was also performed to scrutinize non-specific PCR products. Real-time data were analyzed using Biorad software version 2.1. Relative mRNA expression was calculated using the 2 ^-∆∆CT^ method with GAPDH as a reference. Pearson correlation (*p* < 0.05) was performed to analyze the correlation between the log2fold change of RNA-seq with qPCR data. Significance of gene expression levels (*p* < 0.05) at different days post-amputation tadpole blastemas relative to same-day froglet blastemas was analysed by one-way ANOVA followed by Tukey’s HSD. All statistical analyses were performed, and graphs were prepared with GraphPad Prism 9.3.1.

### In situ hybridisation

Tissue was collected at 1, 3 and 5 dpa and fixed in 4% paraformaldehyde overnight at 4 °C, washed in PBS, brought up a sucrose gradient to 18% sucrose, and embedded in OCT. The blocks were serially sectioned and 8 μm sections were collected. Digoxigenin labelled probes for the *P. maculatus* orthologs of, EPYC, INMT, LEF1 and SALL4 were generated by a two-step PCR amplification of specific DNA sequences and the T7 RNA polymerase promoter (STable 10) as described in Suzuki et al. [67] . For in vitro transcription, the reaction solution with a final volume of 10 μl was prepared with 2–5 ng purified PCR fragments (template DNA) and DIG-RNA-labelling mixture. The reaction was incubated at 37 °C for 6 hours. The RNA probe was precipitated by adding ethanol and cooling at − 20 °C overnight. The RNA probe was isolated and stored at − 80 °C in 30 μl DEPC-H_2_O to inactive before use. The hybridization signals were detected with nitro blue tetrazolium/(5-bromo-4-chloro-3-indolyl-phosphate) (NBT/BCIP 1:200 diluted in AP buffer) in a light-resistant environment for 3–4 min. Sections were counterstained with Fast Green for 1–2 min.

## Supplementary Information

**Additional file 1: S Fig. 1.** Bioinformatics work flow. **S Fig. 2.** Formation of hypomorphic limbs (Red arrows) or no regeneration (White arrow) in *P. maculatus* on limb amputation at NF stage 57–59 tadpoles. A: Reduced shank and incomplete differentiation of toes in the right hindlimb. The left hindlimb is unamputated. B: Reduced shank and incomplete number of toes in the left hindlimb. C: Bent left hindlimb and no regeneration in the right hindlimb. D: Reduced shank and incomplete differentiation of toes in the right hindlimb. Figures not to scale. **S Fig. 3.** Regenerating limb blastemas of tadpole of *P. maculatus* at different intervals post amputation. A: 6 hours post amputation, B: 1 day post amputation, C- 3 days post amputation, D- 5 days post amputation, E- 7 days post amputation, G- 10 days post amputation. we- wound epithelium; b- blastema; st- limb stump; d- developing digit. Blue arrowheads mark the plane of amputation. Bar= A-E=100μm, F=250μm. **S Fig. 4.** Pie-chart showing maximum hits of *P. maculatus* CDS to *Nanorana parkeri*. **S Fig. 5.** GO annotated sequences and WEGO plot of genes of intact froglet hindlimb of *P. maculatus*. **S Fig. 6.** GO annotated sequences and WEGO plot of genes of 3 dpa post amputated froglet hindlimb blastema of *P. maculatus*. **S Fig. 7.** GO annotated sequences and WEGO plot of genes of intact hindlimb of stage 56 tadpoles of *P. maculatus*. **S Fig. 8.** GO annotated sequences and WEGO plot of 3 dpa hindlimb blastema of stage 56 tadpoles of *P. maculatus*. **S Fig. 9.** Summary of the differentially expressed genes of various combinations. Combination 1: Froglet limb vs Tadpole limb. Combination 2: Froglet limb vs 3 dpa froglet limb blastema. Combination 3: Tadpole limb vs 3 dpa tadpole limb blastema. Combination 4: 3 dpa froglet limb blastema vs 3 dpa tadpole limb blastema. **S Fig. 10.** Pearson’s correlation analysis between DGE seq and real time PCR data showing positive correlation. **S Fig. 11.** Differential expression patterns of transcripts by qPCR at different days post amputation tadpole blastemas relative to homeostatic tadpole limb. log2 values of normalized expression of TBFSG (A), KRT10 (B), NNMT-like (C), MMP-18-like (D), FRLP (E), TGFßigh3 (F), MDK (G), IL-8-like (H), EPYC (I), INMT (J), LEF1 (K) of 1,3 and 5 dpa tadpole blastemas relative to homeostatic tadpole limb. Asterisks indicate statistical significance (*: *p*<0.05; **: *p*< 0.01; ***: *p*<0.001; ****: *p*<0.0001). Data are the mean of three biological replicates, each one with three technical replicates. **S Fig. 12.** Differential expression patterns of transcripts by qPCR at different days post amputation froglet blastemas relative to homeostatic froglet limb. log2 values of normalized expression of TBFSG (A), KRT10 (B), NNMT-like (C), MMP-18-like (D), FRLP (E), TGFßigh3 (F), MDK (G), IL-8-like (H), EPYC (I), INMT (J), LEF1 (K) of 1,3 and 5 dpa froglet blastemas relative to homeostatic froglet limb. Asterisks indicate statistical significance (*: *p*<0.05; **: *p*< 0.01; ***: *p*<0.001; ****: *p*<0.0001). Data are the mean of three biological replicates, each one with three technical replicates. **S Fig. 13.** Longitudinal sections through froglet limb with 3 dpa blastema showing GAG+ cells to be NAE+. A: Pentachrome stained section showing GAG+ cells (Black arrowheads), B: NAE stained section showing myeloid cells (Red arrowheads). Black arrows show the plane of amputation. Bars=25μm. we- wound epithelium, st- limb stump. **S Fig. 14.** Myeloid and GAG+ cells in tadpole and froglet limb blastemas of *P. maculatus*. A-C: NAE stained blastemas at different time points post tadpole limb amputation, D-F: NAE stained blastemas at different time points post froglet limb amputation. G: Number of NAE+ cells per mm^2^ of the tadpole blastema at different time points post limb amputation, H: Number of NAE+ cells per mm^2^ of the froglet blastema post limb amputation. (Black arrows indicate myeloid cells). Bars in A-C= 50 μm; D-E=100 μm. **S Fig. 15.** Myeloid cells in the unamputated regions of tadpole limb are INMT+, EPYC+, SALL4+ and LEF1+. A: Pentachrome stained tadpole limb with 5 dpa blastema (Black arrow heads showing GAG+ cells). B: NAE stained serial section of “A” (Blue arrows showing myeloid cells), C-D: *in situ* hybridisation of serial sections of “A” (Black arrows showing hybridisation of GAG+ and NAE+ cells with the genes), E-F: *in situ* hybridisation of 5 dpa blastema of another limb (Black arrows showing hybridisation of GAG+ and NAE+ cells with the genes). Bars= 125μm.

**Additional file 2.**

**Additional file 3.**

**Additional file 4.**

**Additional file 5.**

**Additional file 6.**

**Additional file 7: STable 1**. High quality read statistics of 12 samples. **STable 2.** Transcript, unigenes and CDS Summary. **STable 3.** Group-wise CDS Summary. **STable 4.** GO category distribution of CDS for 4 samples sets. **STable 5.** KEGG Pathway Annotation statistics. **STable 6.** KEGG Pathway classification summary. **STable 7.** Statistics of differentially expressed genes. **STable 8.** Number of GAG+ and NAE+ cells at different time points post amputation of tadpole and froglet limbs. **STable 9.** Primers for DEG’s. **STable 10.** Primers for in situ hybridisation.

## Data Availability

The data that support this study are available from the corresponding author upon reasonable request. RNA sequencing data was uploaded to the NCBI-SRA repository, BioProject ID: PRJNA669113. Source data are included in the manuscript.
