# Supplementary material for: Unravelling the limb regeneration mechanisms of Polypedates maculatus, a sub-tropical frog, by transcriptomics
Source: BMC Genomics. 2023 Mar 16;24:122. doi: 10.1186/s12864-023-09205-8 (PMC10022135; doi:10.1186/s12864-023-09205-8)
Supplement: Supplementary file 1 — Additional file 1: S Fig. 1. Bioinformatics work flow. S Fig. 2. Formation of hypomorphic limbs (Red arrows) or no regeneration (White arrow) in P. maculatus on limb amputation at NF stage 57–59 tadpoles. A: Reduced shank and incomplete differentiation of toes in the right hindlimb. The left hindlimb is unamputated. B: Reduced shank and incomplete number of toes in the left hindlimb. C: Bent left hindlimb and no regeneration in the right hindlimb. D: Reduced shank and incomplete differentiation of toes in the right hindlimb. Figures not to scale. S Fig. 3. Regenerating limb blastemas of tadpole of P. maculatus at different intervals post amputation. A: 6 hours post amputation, B: 1 day post amputation, C- 3 days post amputation, D- 5 days post amputation, E- 7 days post amputation, G- 10 days post amputation. we- wound epithelium; b- blastema; st- limb stump; d- developing digit. Blue arrowheads mark the plane of amputation. Bar= A-E=100μm, F=250μm. S Fig. 4. Pie-chart showing maximum hits of P. maculatus CDS to Nanorana parkeri. S Fig. 5. GO annotated sequences and WEGO plot of genes of intact froglet hindlimb of P. maculatus. S Fig. 6. GO annotated sequences and WEGO plot of genes of 3 dpa post amputated froglet hindlimb blastema of P. maculatus. S Fig. 7. GO annotated sequences and WEGO plot of genes of intact hindlimb of stage 56 tadpoles of P. maculatus. S Fig. 8. GO annotated sequences and WEGO plot of 3 dpa hindlimb blastema of stage 56 tadpoles of P. maculatus. S Fig. 9. Summary of the differentially expressed genes of various combinations. Combination 1: Froglet limb vs Tadpole limb. Combination 2: Froglet limb vs 3 dpa froglet limb blastema. Combination 3: Tadpole limb vs 3 dpa tadpole limb blastema. Combination 4: 3 dpa froglet limb blastema vs 3 dpa tadpole limb blastema. S Fig. 10. Pearson’s correlation analysis between DGE seq and real time PCR data showing positive correlation. S Fig. 11. Differential expression patterns of transcripts by qPCR at diff [file 12864_2023_9205_MOESM1_ESM.pdf]

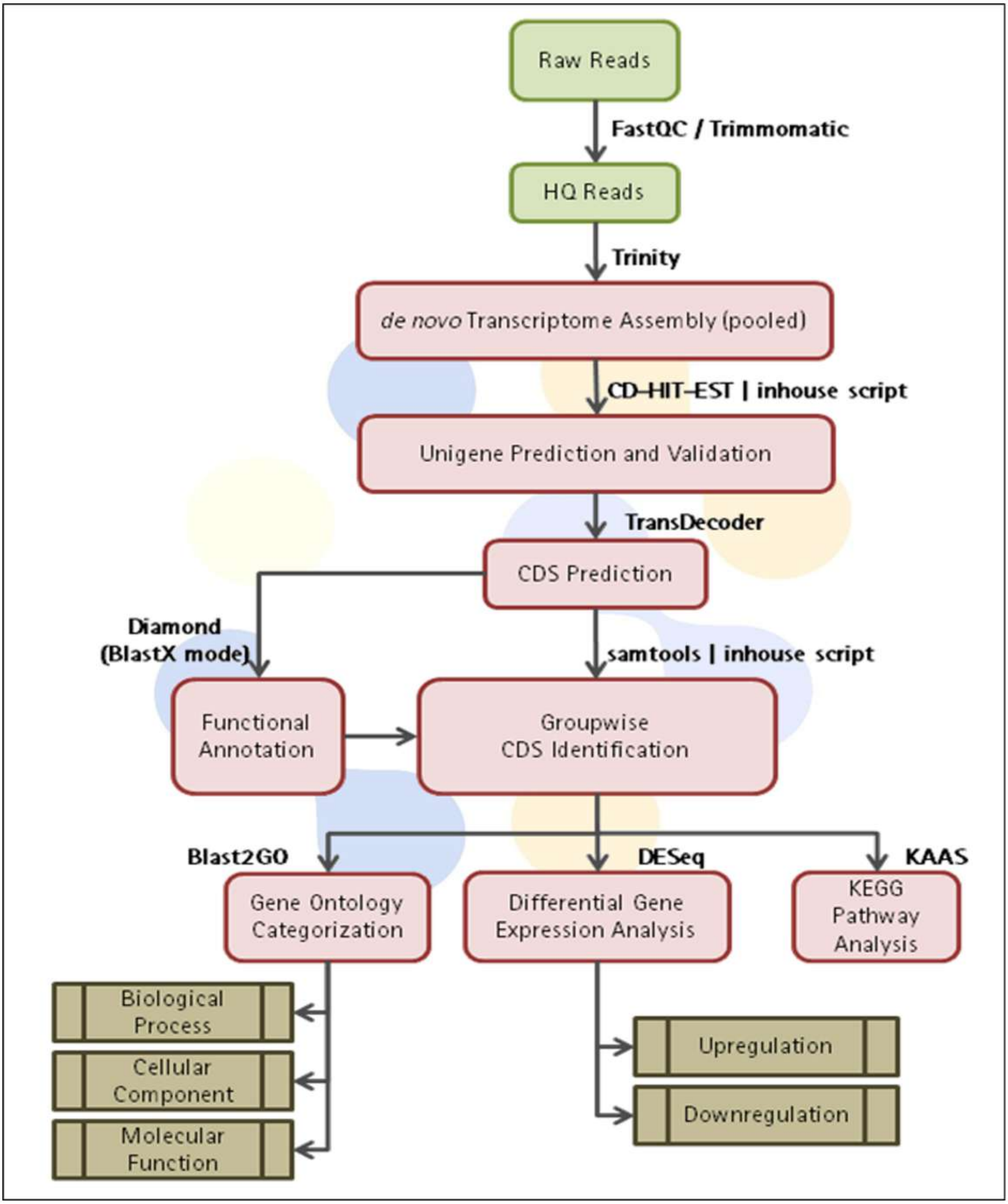

SFig.1. Bioinformatics work flow

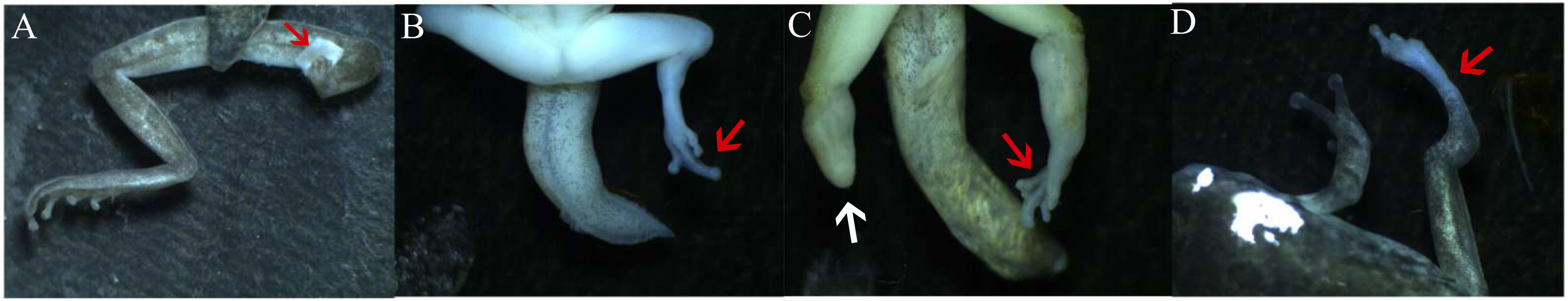

SFig.2. Hypomorphic limbs (Red arrows) or no regeneration (White arrow) in *P. maculatus* on limb amputation at NF stage 57-59 tadpoles.

A: Reduced shank and incomplete differentiation of toes in the right hindlimb.The left hindlimb is unamputated.

B: Reduced shank and incomplete number of toes in the left hindlimb.

C: Bent left hindlimb and no regeneration in the right hindlimb.

D: Reduced shank and incomplete differentiation of toes in the right hindlimb.

Figures not to scale

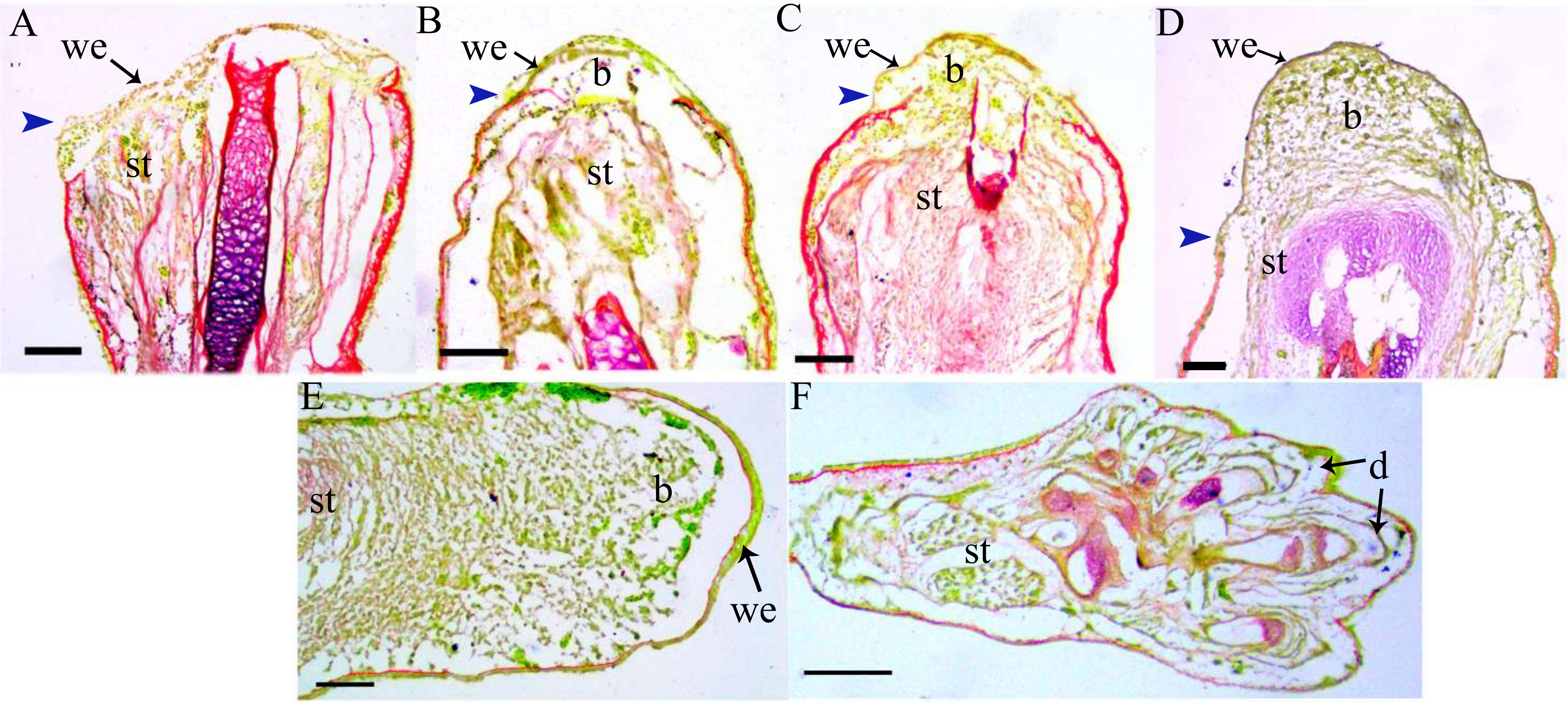

SFig.3. Regenerating limb blastemas of tadpole of *P. maculatus* at different intervals post amputation.
A: 6 hours post amputation, B: 1 day post amputation, C- 3 days post amputation, D- 5 days post amputation, E- 7 days post amputation, G- 10 days post amputation. we- wound epithelium; b- blastema; st- limb stump; d- developing digit
Blue arrowheads mark the plane of amputation
Bar= A-E=100µm,F=250µm

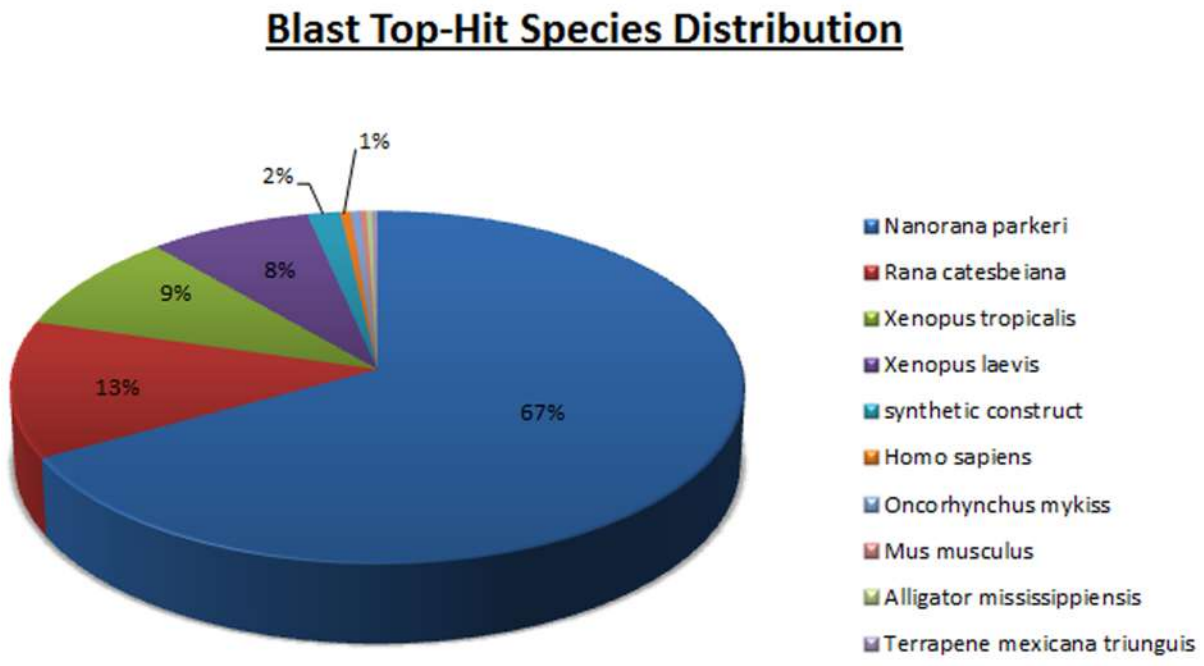

SFig.4. Pie-chart showing maximum hits of *P. maculatus* CDS to *Nanorana parkeri*.

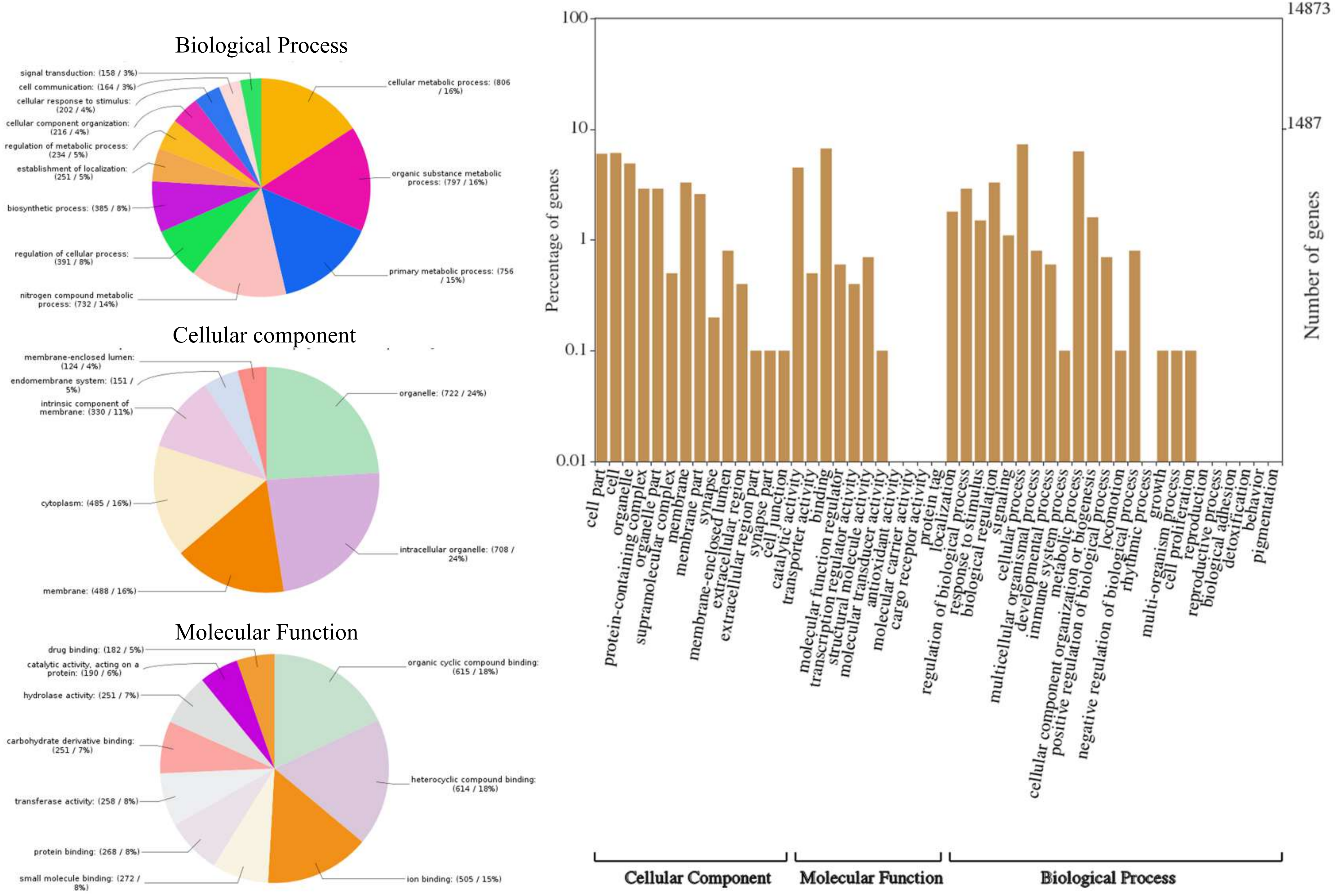

SFig.5. GO annotated sequences and WEGO plot of genes of intact froglet hindlimb of *P. maculatus*.

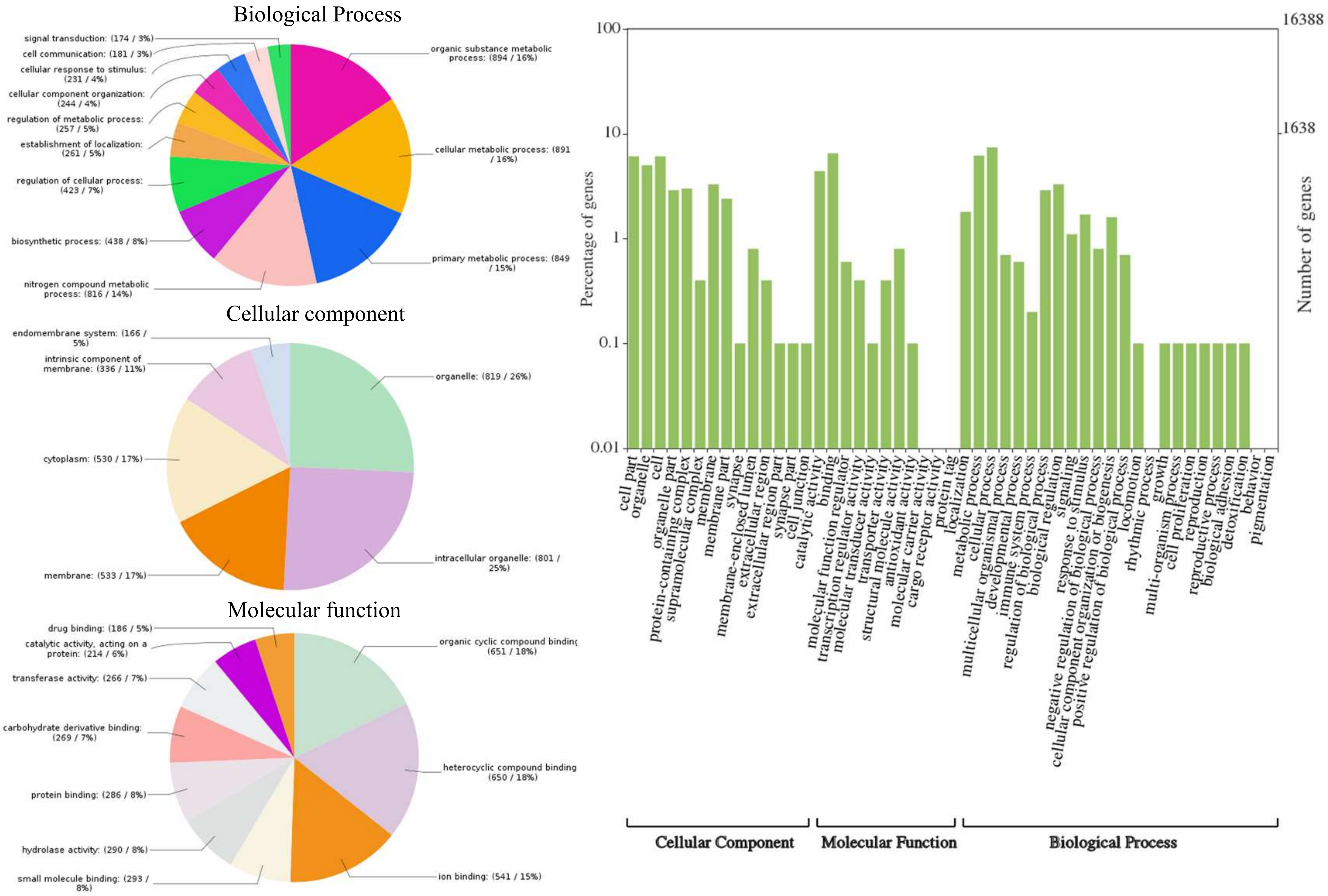

SFig.6. GO annotated sequences and WEGO plot of genes of 3dpa post amputated froglet hindlimb blastema of *P. maculatus*.

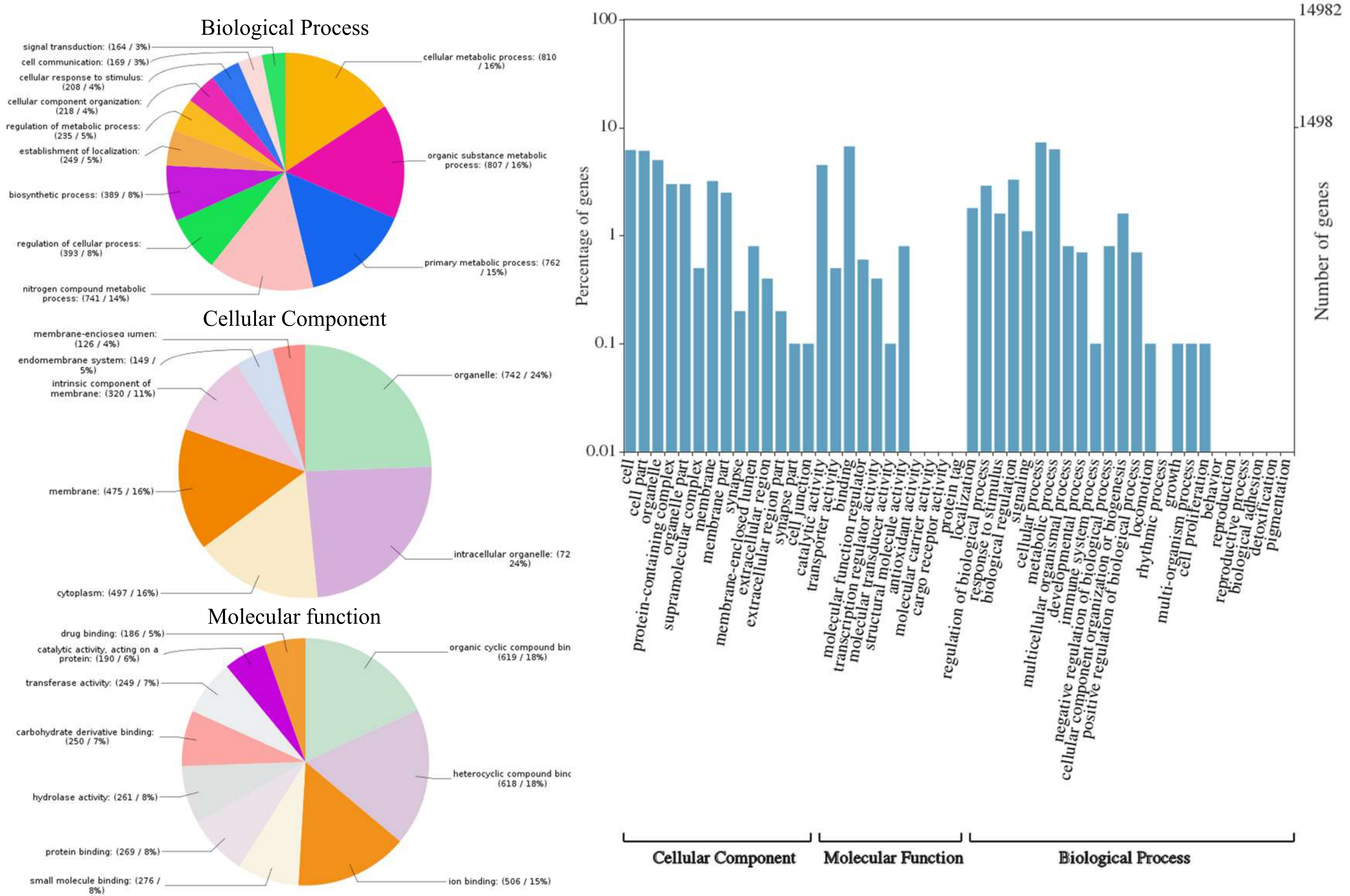

SFig.7. GO annotated sequences and WEGO plot of genes of intact hindlimb of stage 56 tadpoles of *P. maculatus*.

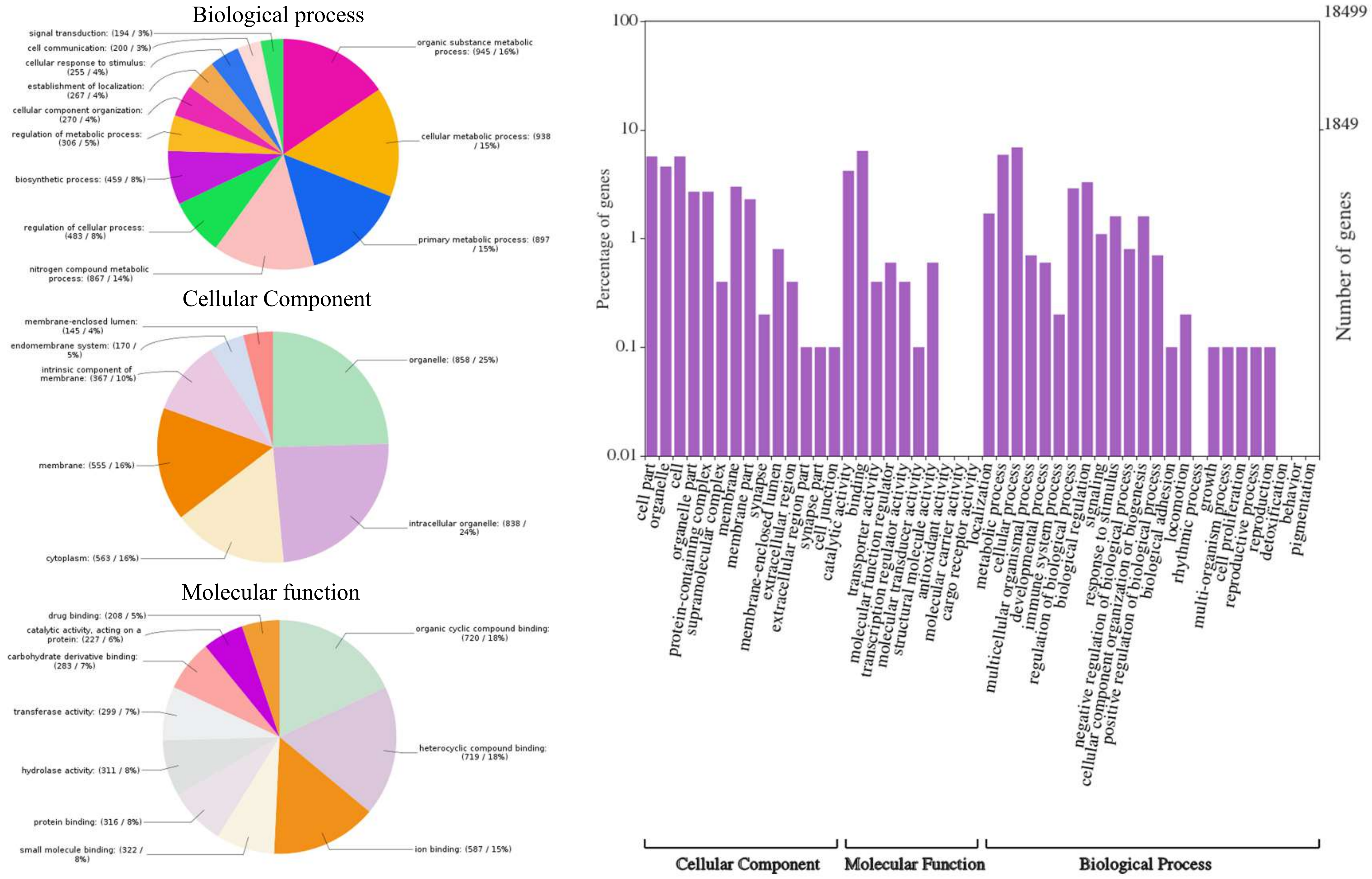

SFig.8. GO annotated sequences and WEGO plot of 3dpa hindlimb blastema of stage 56 tadpoles of *P. maculatus*.

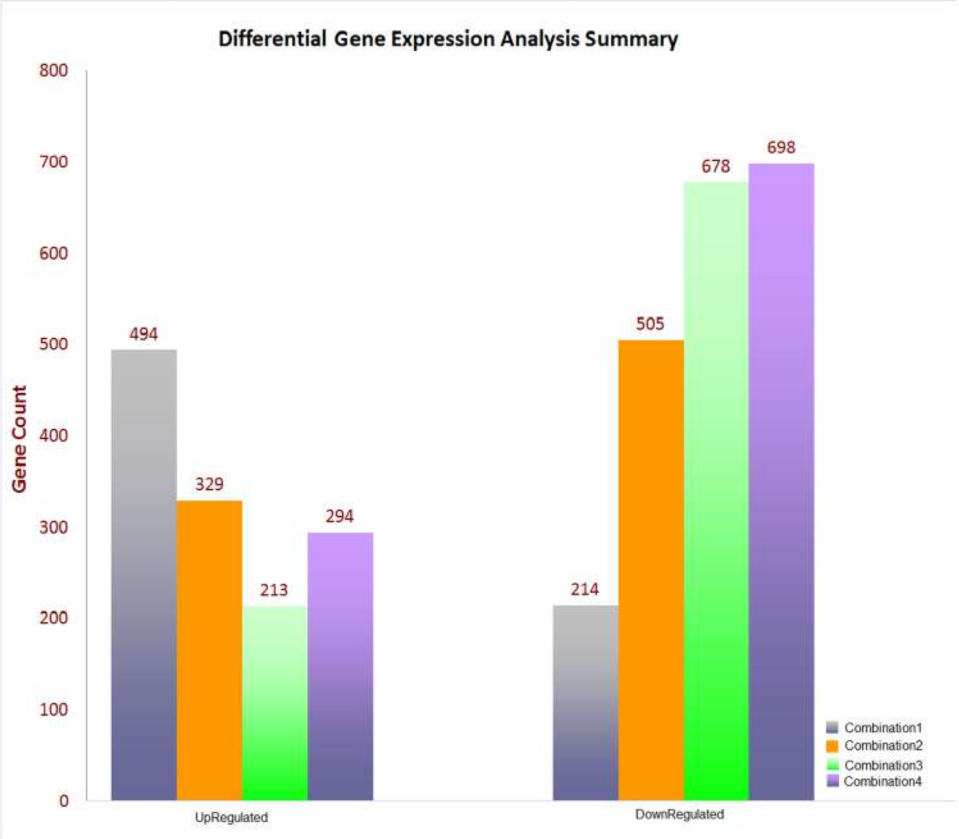

S Fig.9: Summary of the differentially expressed genes of various combinations.
Combination 1: Froglet limb vs Tadpole limb.
Combination 2: Froglet limb vs 3 dpa froglet limb blastema
Combination 3: Tadpole limb vs 3 dpa tadpole limb blastema
Combination 4: 3 dpa froglet limb blastema vs 3 dpa tadpole limb blastema

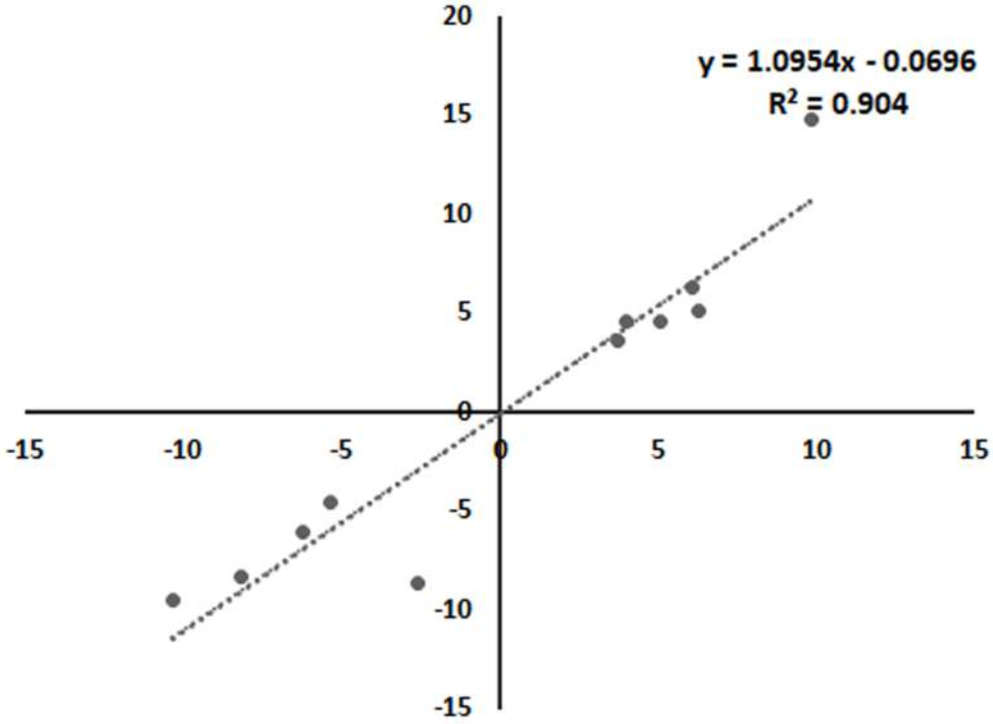

S Fig.10: Pearson's correleation analysis between DGE seq and real time PCR data showing positive correlation.

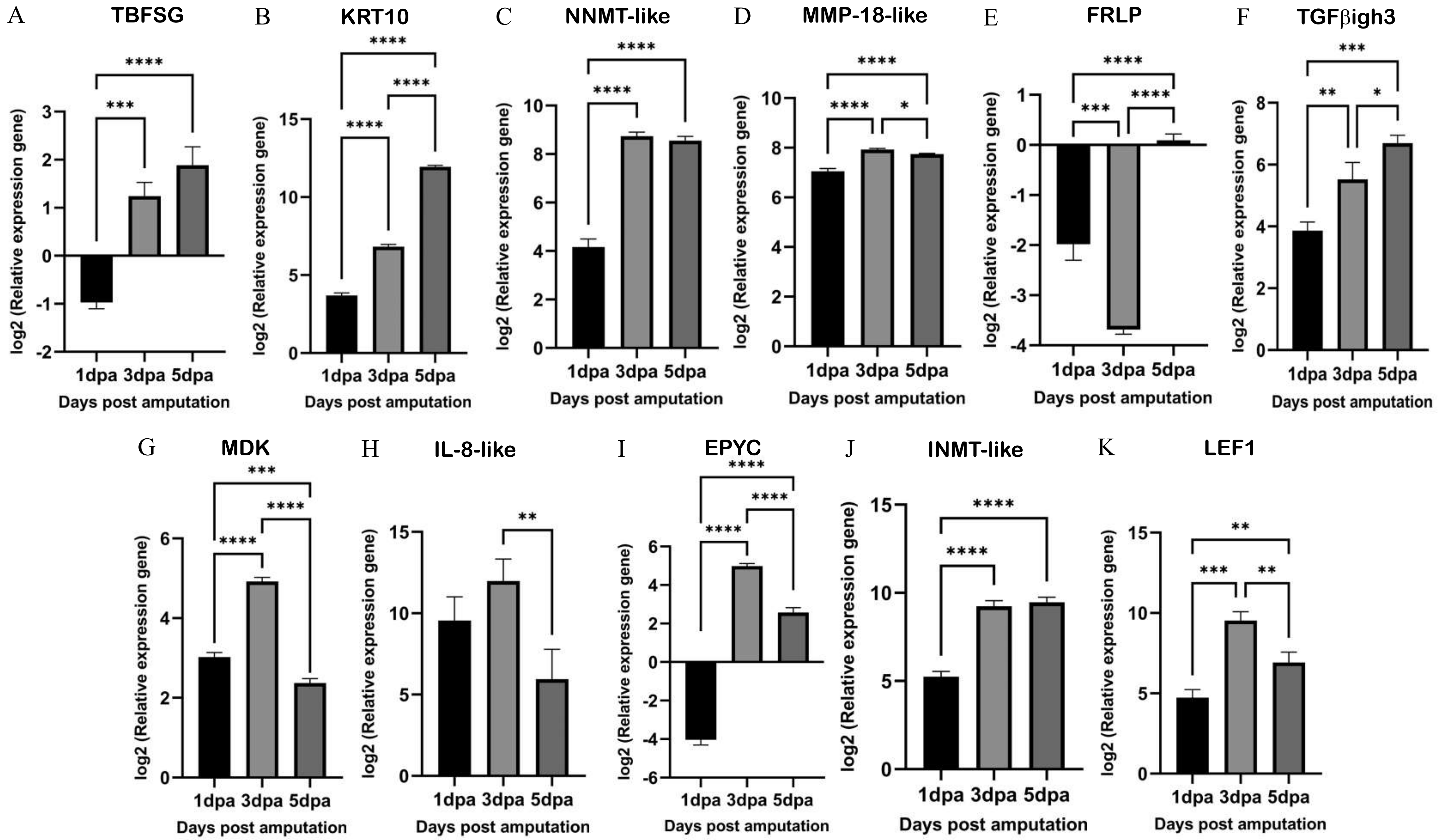

SFig.11. Differential expression patterns of transcripts by qPCR at different days post amputation tadpole blastemas relative to homeostatic tadpole limb. log2 values of normalized expression of TBFSG (A), KRT10 (B), NNMT-like (C), MMP-18-like (D), FRLP (E), TGFβigh3 (F), MDK (G),IL-8-like (H), EPYC (I), INMT (J), LEF1 (K) of 1,3 and 5dpa tadpole blastemas relative to homeostatic tadpole limb. Asterisks indicate statistical significance (*:p<0.05; **: p< 0.01; ***: p<0.001; ****: p<0.0001)
Data are the mean of three biological replicates, each one with three technical replicates.

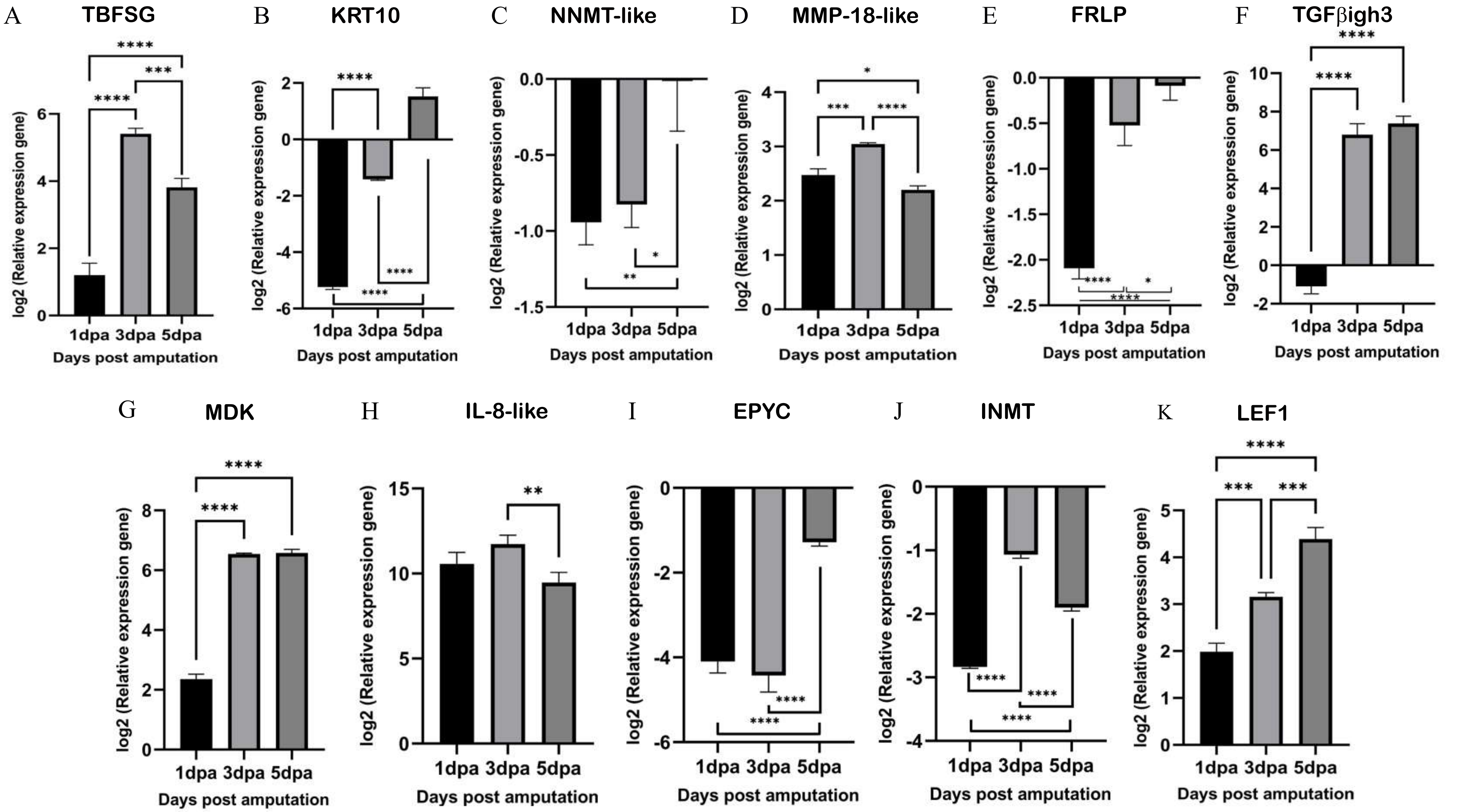

SFig.12. Differential expression patterns of transcripts by qPCR at different days post amputation froglet blastemas relative to homeostatic froglet limb. log2 values of normalized expression of TBFSG (A), KRT10 (B), NNMT-like (C), MMP-18-like (D), FRLP (E), TGFβigh3 (F), MDK (G),IL-8-like (H), EPYC (I), INMT (J), LEF1 (K) of 1, 3 and 5 dpa froglet blastemas relative to homeostatic froglet limb. Asterisks indicate statistical significance (*:$p<0.05$; **: $p< 0.01$; ***: $p<0.001$; ****: $p<0.0001$)

Data are the mean of three biological replicates, each one with three technical replicates.

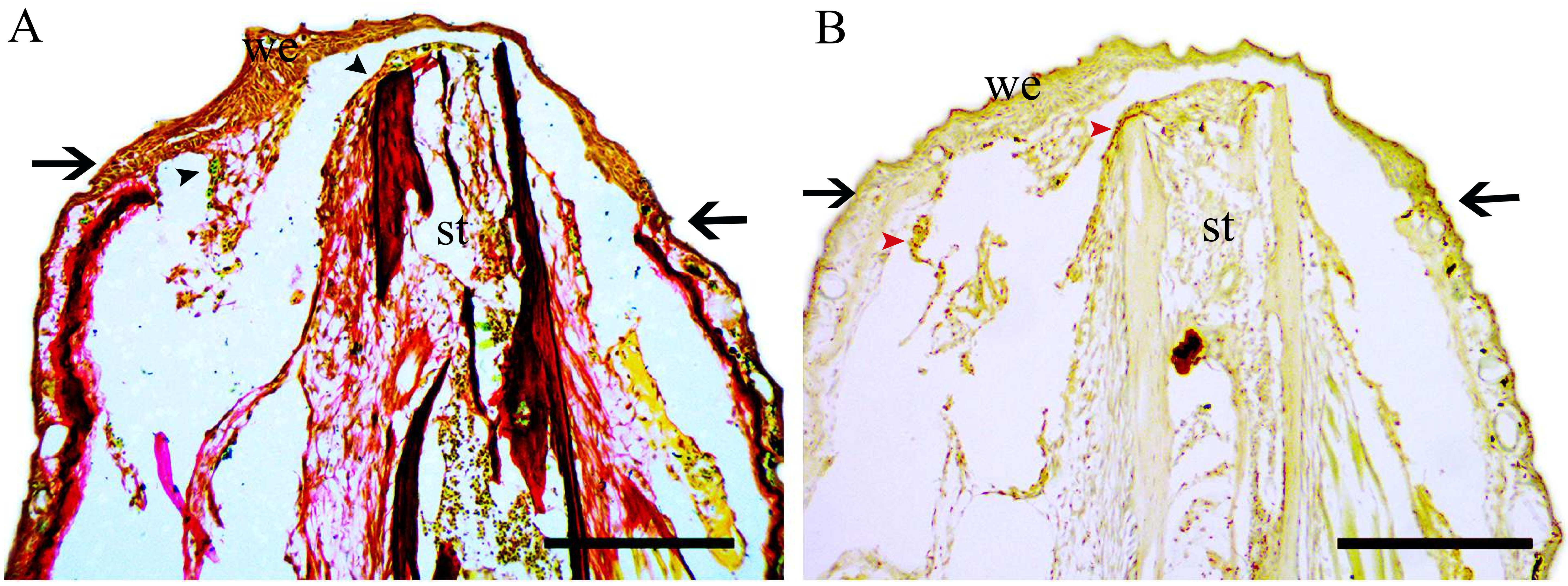

SFig.13. Longitudinal sections through froglet limb with 3 dpa blastema showing GAG+ cells to be NAE+. A: Pentachrome stained section showing GAG+ cells (Black arrowheads), B: NAE stained section showing myeloid cells (Red arrow heads). Black arrows show the plane of amputation. Bars=25µm.
we- wound epithelium, st- limb stump.

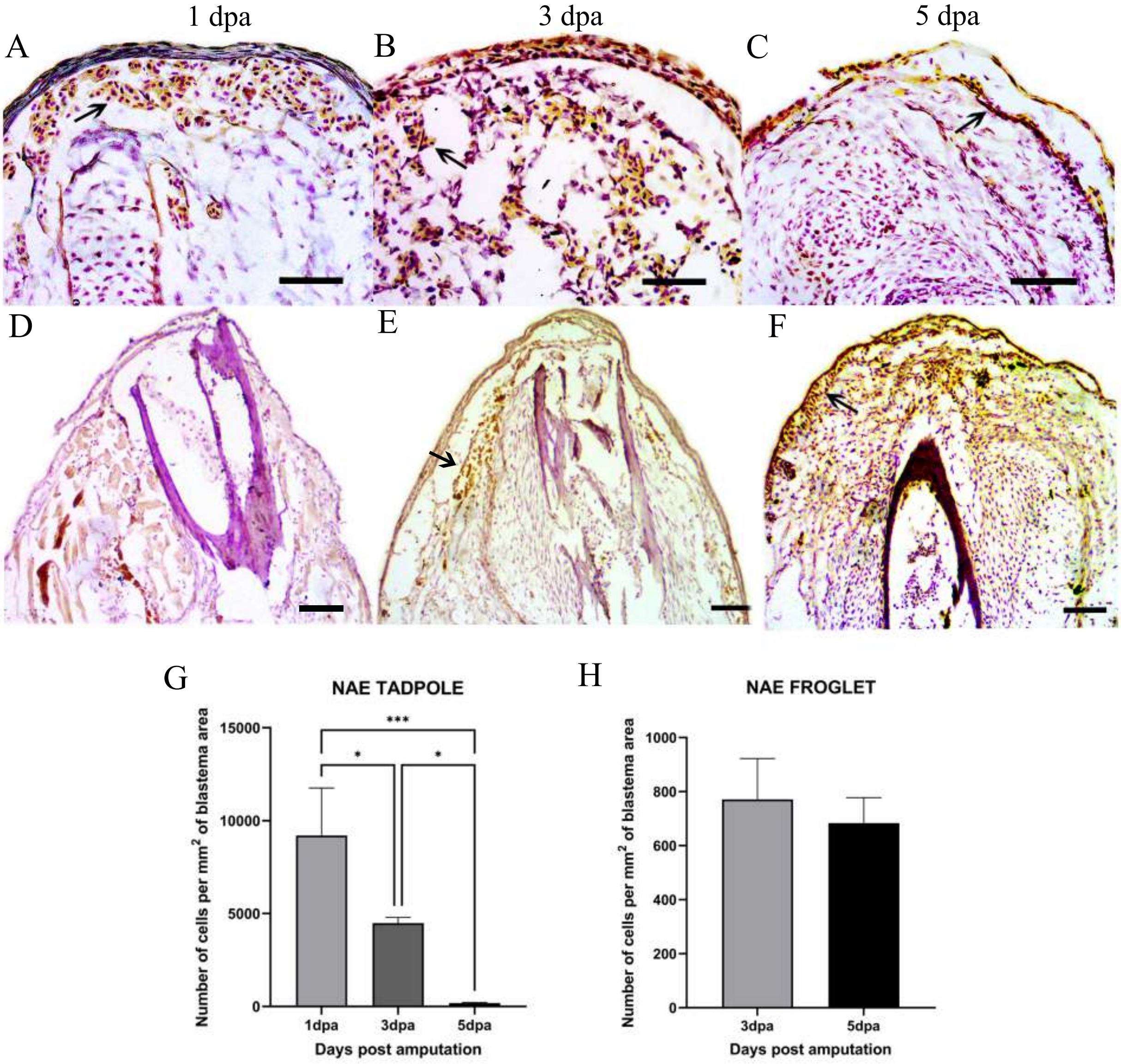

SFig.14. Myeloid and GAG+ cells in tadpole and froglet limb blastemas of *P. maculatus*. .
A-C: NAE stained blastemas at different time points post tadpole limb amputation,
D-F: NAE stained blastemas at different time points post froglet limb amputation.
G: Number of NAE+ cells per mm^2 of the tadpole blastema at different time points post limb amputation,H:Number of NAE+ cells per mm^2 of the froglet blastema post limb amputation. (Black arrows indicate myeloid cells)
Bars in A-C= 50 μm; D-E=100 μm.

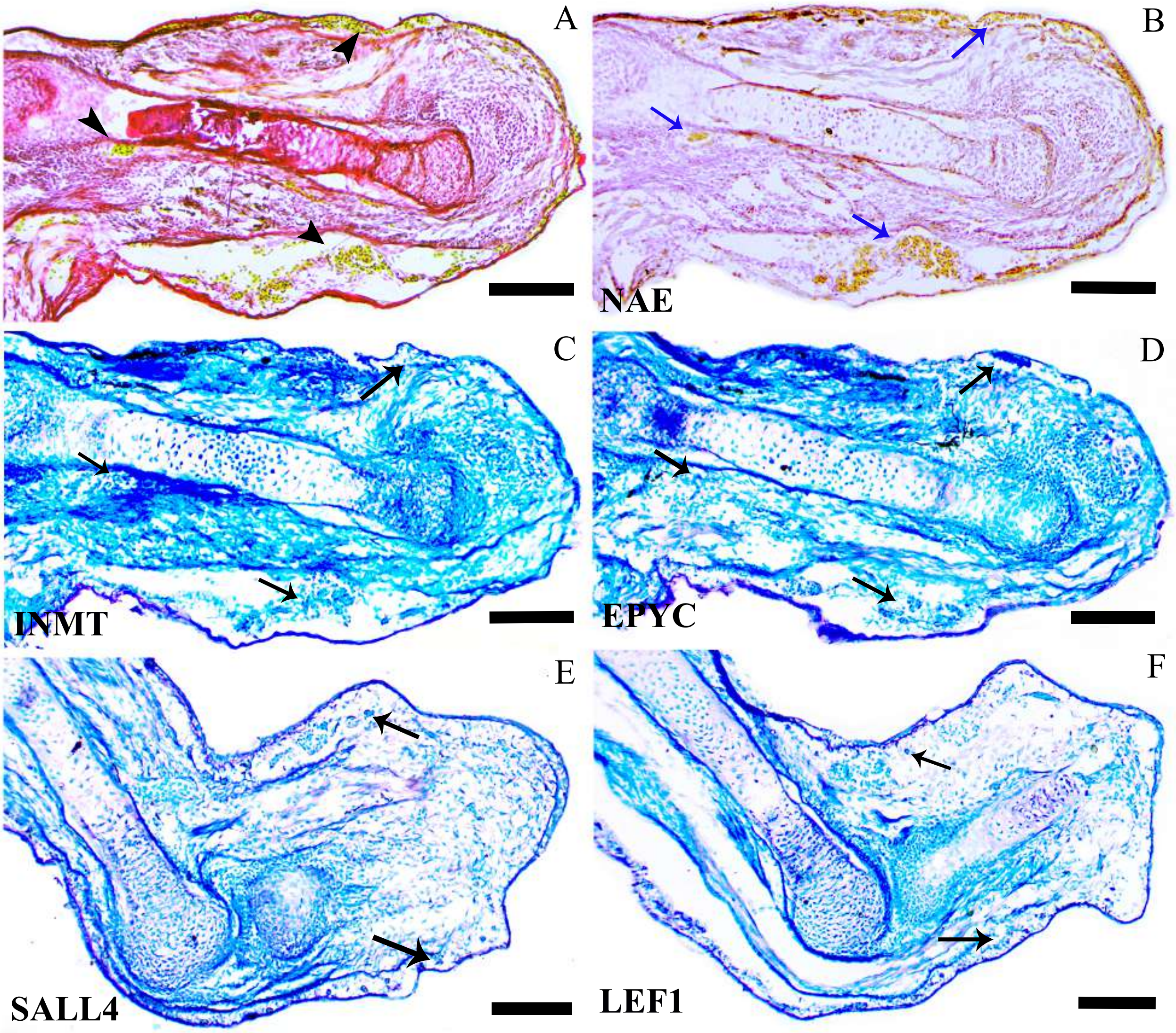

SFig.15. Myeloid cells in the unamputated regions of tadpole limb are INMT+, EPYC+, SALL4+ and LEF1+. A: Pentachrome stained tadpole limb with 5 dpa blastema (Black arrow heads showing GAG+ cells). b: NAE stained serial section of "A" (Blue arrows showing myeloid cells), C-D: *in situ* hybridisation of serial sections of "A" (Black arrows showing hybridisation of GAG+and NAE+ cells with the genes), E-F: *in situ* hybridisation of 5 dpa blastema of another limb (Black arrows showing hybridisation of GAG+and NAE+ cells with the genes). Bars= 125μm
