# Supplementary material for: Unravelling the limb regeneration mechanisms of Polypedates maculatus, a sub-tropical frog, by transcriptomics
Source: BMC Genomics. 2023 Mar 16;24:122. doi: 10.1186/s12864-023-09205-8 (PMC10022135; doi:10.1186/s12864-023-09205-8)
Supplement: Supplementary file 7 — Additional file 7: STable 1. High quality read statistics of 12 samples. STable 2. Transcript, unigenes and CDS Summary. STable 3. Group-wise CDS Summary. STable 4. GO category distribution of CDS for 4 samples sets. STable 5. KEGG Pathway Annotation statistics. STable 6. KEGG Pathway classification summary. STable 7. Statistics of differentially expressed genes. STable 8. Number of GAG+ and NAE+ cells at different time points post amputation of tadpole and froglet limbs. STable 9. Primers for DEG’s. STable 10. Primers for in situ hybridisation. [file 12864_2023_9205_MOESM7_ESM.docx]

**STable 1: High quality read statistics of 12 samples**

| SL Num | Sample Name | No. of PE Reads | Number of bases | Total data inGb |
| --- | --- | --- | --- | --- |
| 1 | Group1- A  (Froglet limb) | 30,807,418 | 4,625,983,625 | 4.63 |
| 2 | Group1-B  (Froglet limb) | 22,099,568 | 3,321,600,885 | 3.32 |
| 3 | Group1-C  (Froglet limb) | 24,020,350 | 3,610,712,549 | 3.61 |
| 4 | Group2-A  (3dpa froglet blastema) | 30,830,467 | 4,631,082,577 | 4.63 |
| 5 | Group2-B  (3dpa froglet blastema) | 25,956.152 | 3,901,168,747 | 3.90 |
| 6 | Group2-C  (3dpa froglet blastema) | 22,850,601 | 3,434,498,750 | 3.43 |
| 7 | Group3-A  (Tadpole limb) | 35,783,757 | 5,373,032,785 | 5.37 |
| 8 | Group3-B  (Tadpole limb) | 26,220,005 | 3,927,781,580 | 3.9 |
| 9 | Group3-C  (Tadpole limb) | 28,491,519 | 4,267,822,067 | 4.3 |
| 10 | Group4-A  (3dpa tadpole blastema) | 36,232,175 | 5,442,703,208 | 5.44 |
| 11 | Group4-B  (3dpa tadpole blastema) | 24,788,115 | 3,724,636,285 | 3.72 |
| 12 | Group4-C  (3dpa tadpole blastema) | 25,398,177 | 3,817,477,112 | 3.82 |

**STable 2: Transcript, unigenes and CDS Summary**

| Description |  |
| --- | --- |
| No. of Transcripts | 240,159 |
| Total transcript length (bp) | 184,227,931 |
| N50 (bp) | 1,439 |
| Maximum transcript length (bp) | 86,576 |
| Minimum transcript length (bp) | 201 |
| Mean transcript length (bp) | ~767.1 |
| No. of Unigenes | 109,674 |
| Total unigene length (bp) | 102,863,734 |
| N50 (bp) | 1,652 |
| Maximum unigene length (bp) | 86,576 |
| Minimum unigene length (bp) | 201 |
| Mean unigene lenth (bp) | 938 |
| No. Of CDS | 28,427 |
| Total CDS length (bp) | 31,844,553 |
| N50 (bp) | 1,581 |
| Maximum CDS length (bp) | 86,259 |
| Minimum CDS length (bp) | 255 |
| No. of CDS with Blast Hit | 20,934 |
| No. of CDS without Blast Hit | 7,493 |

**STable 3: Group-wise CDS Summary**

| Sl. No. | Sample group Name | No. of CDS | Total CDS length (bp) | Maximum CDS length (bp) | Minimum CDS length (bp) | Mean CDS length (bp) |
| --- | --- | --- | --- | --- | --- | --- |
| 1 | Group1  (Froglet limb) | 14,873 | 14,900,691 | 13,683 | 255 | 1,002 |
| 2 | Group2  (3dpa froglet blastema) | 16,388 | 16,435,752 | 13,683 | 255 | 1,003 |
| 3 | Group3  (Tadpole limb) | 14,982 | 14,122,563 | 13,683 | 255 | 943 |
| 4 | Group4  (3dpa tadpole blastema) | 18,499 | 19,954,011 | 13,683 | 261 | 1,079 |

**STable 4: GO category distribution of CDS for 4 samples sets**

| Sl. no. | Sample group Name | No. of CDS | Total number of annotated CDS | Biological Process | Cellular Component | Molecular Function |
| --- | --- | --- | --- | --- | --- | --- |
| 1 | Group1  (Froglet limb) | 14,873 | 1,930 | 1,392 | 1,237 | 1,458 |
| 2 | Group2  (3dpa froglet blastema) | 16,388 | 2,122 | 1,526 | 1,366 | 1,575 |
| 3 | Group3  (Tadpole limb) | 14,982 | 1,952 | 1,407 | 1,257 | 1,468 |
| 4 | Group4  (3dpa tadpole blastema) | 18,499 | 2,252 | 1,618 | 1,437 | 1,695 |

**STable 5: KEGG Pathway Annotation statistics**

| Sl. No. | Sample group Name | CDS | Number of KEGG Annotated CDS |
| --- | --- | --- | --- |
| 1 | Group1  (Froglet limb) | 14,873 | 6,388 |
| 2 | Group2  (3dpa froglet blastema) | 16,388 | 6,940 |
| 3 | Group3  (Tadpole limb) | 14,982 | 6,067 |
| 4 | Group4  (3dpa tadpole blastema) | 18,499 | 7,400 |

**STable 6: KEGG Pathway classification summary**

| **Pathways** | **Group 1** | **Group 2** | **Group 3** | **Group 4** |
| --- | --- | --- | --- | --- |
| **Metabolism** | | | | |
| Carbohydrate metabolism | 222 | 239 | 219 | 248 |
| Energy metabolism | 148 | 148 | 148 | 149 |
| Lipid metabolism | 239 | 267 | 241 | 262 |
| Nucleotide metabolism | 88 | 102 | 86 | 108 |
| Amino acid metabolism | 175 | 198 | 173 | 213 |
| Metabolism of other amino acids | 85 | 97 | 90 | 101 |
| Metabolism of cofactors and vitamins | 147 | 158 | 148 | 165 |
| **Genetic Information Processing** | | | | |
| Transcription | 189 | 199 | 191 | 208 |
| Translation | 369 | 395 | 366 | 421 |
| Folding, sorting and degradation | 353 | 374 | 350 | 388 |
| Replication and repair | 92 | 121 | 95 | 142 |
| **Environmental Information Processing** | | | | |
| Membrane transport | 14 | 13 | 11 | 14 |
| Signal transduction | 841 | 933 | 776 | 1031 |
| Signaling molecules and interacrion | 171 | 218 | 158 | 254 |
| **Cellular Processes** | | | | |
| Transport and catabolism | 504 | 558 | 471 | 569 |
| Cell growth and death | 344 | 409 | 339 | 437 |
| Cellular community- eukaryotes | 298 | 314 | 268 | 350 |
| Cell motility | 104 | 116 | 95 | 124 |
| **Organismal Systems** | | | | |
| Immune system | 425 | 501 | 380 | 510 |
| Endocrine system | 439 | 448 | 412 | 488 |
| Circulatory system | 137 | 125 | 125 | 131 |
| Digestive system | 182 | 182 | 171 | 190 |
| Excretory system | 97 | 85 | 94 | 84 |
| Nervous system | 235 | 230 | 214 | 246 |
| Sensory system | 42 | 40 | 35 | 48 |
| Development and regeneration | 156 | 182 | 136 | 200 |
| Aging | 106 | 104 | 98 | 114 |
| Environmental adaptation | 186 | 184 | 177 | 205 |

**STable 7: Statistics of differentially expressed genes**

| Description | Commonly Expressed | Upregulated | Downregulated |
| --- | --- | --- | --- |
| Combination 1  (Froglet limb vs Tadpole limb) | 13,550 | 494 | 214 |
| Combination 2  (Froglet limb vs 3dpa froglet blastema ) | 13,122 | 329 | 505 |
| Combination 3  (Tadpole limb vs 3dpa tadpole blastema) | 13,387 | 213 | 678 |
| Combination 4  (3dpa froglet blastema vs 3dpa tadpole blastema) | 14,874 | 294 | 698 |

**STable 8: Number of GAG+ and NAE+ cells at different time points post amputation of tadpole and froglet limbs.**

| **Time points of blastema** | **Number of GAG+ cells** | **Number of NAE+ cells** |
| --- | --- | --- |
| 1dpa tadpole | 170.667 ± 2.52* | 111.334 ± 7.23 |
| 3dpa tadpole | 205.334 ± 6.03 | 187.334 ± 7.09 |
| 5dpa tadpole | 283 ± 6.25 | 243 ± 13.52 |
| 3dpa froglet | 161.667 ± 12.58 | 125 ± 9.6 |
| 5dpa froglet | 75.667 ± 13.20 | 61.334 ± 11.06 |

*Number given as Mean ± Standard deviation.

**STable 9: Primers for DEG’s**

| LEF1 F | CACATACAGCGACGAGCACT |
| --- | --- |
| LEF1 R | ATACCAGCCAAGAGGTGGTG |
| SALL 4 F | AAACCTTTTGCCTGCACGAT |
| SALL 4 R | ACTGGTTCCACACAACAGGA |
| IL-8-like F | TCAGTGGGAGGGGATTTGC |
| IL-8-like R | CAAATTCTGAGGCCACGCTT |
| INMT F | TCTGGAAAGGAGGAAATGGTCT |
| INMT R | TGGAAGACATTGGAGGCAGA |
| TBFSG F | GTCTATCCCGTCTGAGTGCA |
| TBFSG R | CAAGACCACCACCTACACCT |
| EPYC F | GTTTCCTCGATGCCAGCAAA |
| EPYC R | CGAGGGACCTTCGAGCATAT |
| FRLP F | TACGGTGCAGTGTAAGGAGG |
| FRLP R | CCTGTTGGCTCACACACTTG |
| KRT 10 F | GCCCAACACCAGATCCAAAT |
| KRT 10 R | CCGGTACTGCTGCTTTCATC |
| MMP18-like F | AATCTGTTTCTGGTGGCTGC |
| MMP18-like R | CATTTGGAAGGGGTGGTTGG |
| MDK F | TGCGAGCTTTGTGTGTCATC |
| MDK R | TTTACATGTCCCCTCCCGAG |
| Myosin F | AAAAGCAAAGAAGAGGGCGG |
| Myosin R | GCAGCGAATGTGTCTCTCAG |
| Aquaporin F | GTTAACCTGCCAGCCAACAA |
| Aquaporin R | ATAAGTGCCGGACCAAAGGA |
| TGFβigh3 F | GCAATGAGGCATGGAAAGCT |
| TGFβigh3 R | TTGTTGCATGATGGTCAGCC |
| NNMT-like F | TTGGCTCCTTCATCCACCAT |
| NNMT-like R | TGGCCGCCTTCAGAGATAAT |
| Glutaredoxin F | CCATCCTTAAGCAGCACACC |
| Glutaredoxin R | GTCTCACCAGCTCTCCACTT |
| Hemoglobin F | GGCACGGAGGAAATGTTCTC |
| Hemoglobin R | GGCGTTGAATTCCTTGGGGA |

**STable 10: Primers for *in situ* hybridisation**

| EPYC F | GTTTCCTCGATGCCAGCAAA |
| --- | --- |
| EPYC R | CGAGGGACCTTCGAGCATAT |
| EX+T7+  EPYC R | CAGTGAATTGTAATACGACTCACTATAGGGAGACGAGGGACCTTCGAGCATAT |
| SALL4 F | AAACCTTTTGCCTGCACGAT |
| SALL4 R | ACTGGTTCCACACAACAGGA |
| EX+T7+  SALL4 R | CAGTGAATTGTAATACGACTCACTATAGGGAGAACTGGTTCCACACAACAGGA |
| LEF1 F | CACATACAGCGACGAGCACT |
| LEF1 R | ATACCAGCCAAGAGGTGGTG |
| EX+T7+  LEF1 R | CAGTGAATTGTAATACGACTCACTATAGGGAGAATACCAGCCAAGAGGTGGTG |
| INMT F | TCTGGAAAGGAGGAAATGGTCT |
| INMT R | TGGAAGACATTGGAGGCAGA |
| EX+T7+  INMT R | CAGTGAATTGTAATACGACTCACTATAGGGAGATGGAAGACATTGGAGGCAGA |
